# Nuclear factor IX promotes glioblastoma development through transcriptional activation of Ezrin

**DOI:** 10.1038/s41389-020-0223-2

**Published:** 2020-04-14

**Authors:** Zhuohao Liu, Ruixiang Ge, Jiayi Zhou, Xinzhi Yang, Kenneth King-yip Cheng, Jingli Tao, Dinglan Wu, Jie Mao

**Affiliations:** 1 Department of Neurosurgery, Shenzhen Hospital, Southern Medical University, Shenzhen, Guangdong China; 2 Department of Neurosurgery, Yijishan Hospital, Wannan Medical College, Wuhu, Anhui China; 3 Shenzhen Key Laboratory of Viral Oncology, The Clinical Innovation & Research Centre, Shenzhen Hospital, Southern Medical University, Shenzhen, Guangdong China; 4 Department of Health Technology and Informatics, The Hong Kong Polytechnic University, Hong Kong, China; 5 Department of Pathology, Shenzhen Hospital, Southern Medical University, Shenzhen, Guangdong China

**Keywords:** CNS cancer, Cancer therapy

## Abstract

Enhanced migration is pivotal for the malignant development of glioblastoma (GBM), but the underlying molecular mechanism that modulates the migration of the GBM cells remains obscure. Here we show that nuclear factor IX (NFIX) is significantly upregulated in human GBM lesions compared with normal or low-grade gliomas. NFIX deficiency impairs the migration of GBM cells and inhibits the tumor growth in the hippocampus of immunodeficient nude mice. Mechanistically, NFIX silencing suppresses the expression of Ezrin, a protein that crosslinks actin cytoskeleton and plasma membrane, which is also positively correlated with GBM malignancy. NFIX depletion induced migration inhibition of GBM cells can be rescued by the replenishment of Ezrin. Furthermore, we identify a NFIX response element (RE) between −840 and −825 bp in the promoter region of the *Ezrin* gene. Altogether, our findings show, for the first time that NFIX can transcriptionally upregulate the expression of Ezrin and contribute to the enhanced migration of GBM cells, suggesting that NFIX is a potential target for GBM therapy.

## Introduction

Glioblastomas (GBM), defined as grade IV astrocytomas based on the WHO classification, are the most aggressive and malignant brain tumors in adults^1,2^. Despite recent advances in therapeutic intervention, prognosis for GBM patients remains poor, with around 15 months median survival time^3^. Generally, standard surgical resection followed by radiotherapy and chemotherapy, cannot significantly improve overall survival time due to its highly infiltrative property and tumor reoccurrences^4,5^. Although many efforts have been made to investigate the molecular machinery underlying invasion and migration of GBM, the transcription factors responsible for the pathogenesis are still poorly understood. Thus, identification of these transcription factors might provide us new therapeutic targets to treat GBM.

The nuclear factor I (NFI) is a family of transcription factors, which have conserved recognition sites (TGGC(N_5_)GCCA) that are enriched in many brain-specific promoters^6,7^. NFI can regulate the gene expression of abundant GBM related genes, including *brain fatty acid-binding protein* (*B-FABP*), *glial fibrillary acidic protein* (*GFAP*) and notch effector gene *HEY1*^8–10^. The NFI family consists of four members including NFIA, NFIB, NFIC and NFIX^11^. Studies have attempted to explore the functional roles of NFI family in GBM. In particular, NFIA is highly expressed in human GBM when compared with normal brain tissue^12–14^. NFIA plays a tumor-promoting role in GBM development as shown by enhancing GBM cells growth, proliferation and migration^13^. However, the roles of NFIB in GBM were found to be controversial. Two research teams illustrated that NFIB exerts an anti-tumor effect in GBM^15,16^. On the contrary, Li et al. recently demonstrated that NFIB, as a downstream target of miR-346, promotes the proliferation of GBM cells^17^.

Unlike NFIA and NFIB, our understanding on NFIX in GBM development is limited. NFIX has been shown to play a role in regulation of muscle development, hematopoiesis, and also be involved in the development of prostate cancer, esophageal squamous cell carcinoma and colorectal cancer^18–23^. In addition, NFIX is highly expressed in nervous systems and is crucial for brain development^24–26^. However, the gene expression of NFIX in GBM and the role of NFIX in the regulation of GBM development are still unclear.

In this study, we discovered that NFIX is significantly upregulated in human GBM. To gain insight into the functional significance of NFIX in GBM, we orthotopically implanted NFIX deficient GBM cells into the hippocampus of nude mice and we demonstrate that NFIX exerts tumor-promoting role in malignant GBM development. NFIX deficiency impairs GBM cell migration and attenuates malignant progression of GBM in an Ezrin-dependent manner. Importantly, this effect is mediated through the specific NFIX-recognition sequences in the promoter of *Ezrin*. Our results highlighted a crucial role of the NFIX-Ezrin axis in regulating the migration of GBM cells during malignant GBM progression.

## Results

### NFIX is upregulated in human GBM

**Fig. 1:**
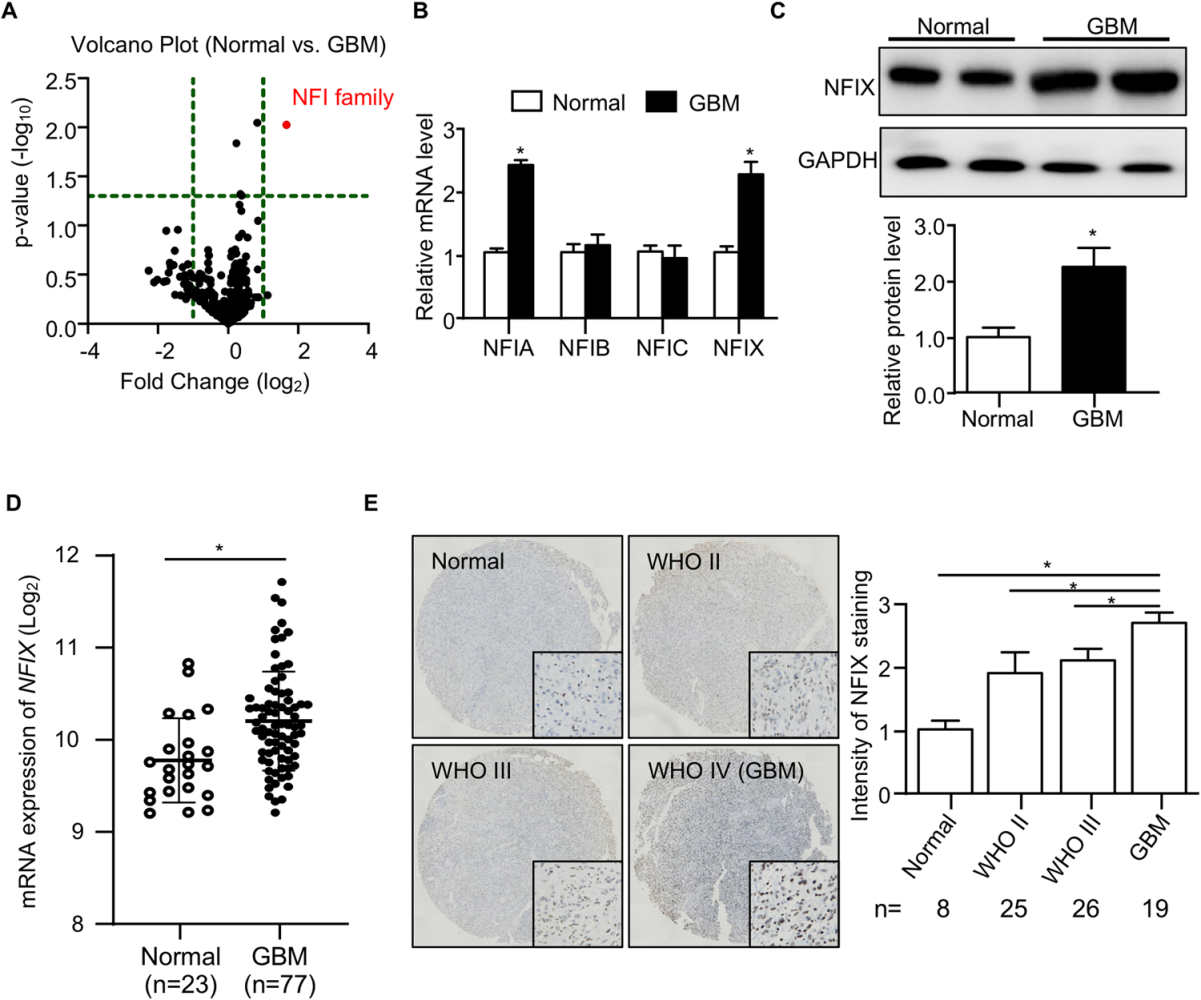
NFIX is upregulated in human GBM. **a**–**c** Human GBM tissues and normal brain tissues were used. **a** Valcano plot of transcription factors identified by oligonucleotide array-based transcription factor assay (*n* = 5). The vertical lines correspond to 1.5-fold up and down, respectively, and the horizontal line stands for a *p*-value of 0.05. **b** Relative mRNA levels of *NFIA, NFIB, NFIC*, and *NFIX* normalized with *GAPDH* in human GBM tissues and normal brain tissues (*n* = 5). **c** Immunoblotting analysis of *NFIX* and GAPDH in human GBM tissues and normal brain tissues. Representative images are shown. The bar chart is a relative expression level of NFIX normalized with GAPDH (*n* = 5). **d** *NFIX* mRNA level in human normal brain tissues and GBM (GSE4290; *n* = 23 for normal brain tissue and *n* = 77 for GBM). **e** IHC staining with the NFIX antibody in human glioma tissue microarray (*n* = 8 for normal brain tissue, *n* = 25 for WHO II, *n* = 26 for WHO III and *n* = 19 for GBM). All data are represented as the mean ± s.e.m. *p < 0.05, Normal vs. GBM group (Student’s *t* test).

Our oligonucleotide array-based transcription factor assay of human GBM tissues and normal brain tissues identified 345 transcription factors. Volcano plot analysis revealed that NFI family changed significantly in GBM (Fig. 1a), indicating that NFI family may play a role in the progression of GBM. As there are four members of NFI family (NFIA, NFIB, NFIC, and NFIX), we next examined which member of the NFI family contributes to the increased expression of NFI. Quantitative PCR (QPCR) analysis illustrated that the mRNA abundance of *NFIA* and *NFIX* were significantly increased in human GBM tissues (Fig. 1b). Since the roles of NFIA in GBM development have been well investigated^12,13^, we aimed to focus on NFIX in this study. Consistent to the mRNA expression, the protein level of NFIX was upregulated in GBM tissues when compared with normal brain tissues (Fig. 1c). We next explored the expression of NFIX in GBM from published human dataset (GSE4290). Expression of NFIX was significantly increased in GBM compared with normal brain tissues (Fig. 1d), which was consistent with our results. To further confirm the NFIX expression in GBM, we performed IHC staining in tissue microarray (TMA). IHC staining showed that the NFIX was increased in low-grade glioma samples, and even further enriched in the GBM (Fig. 1e). These findings indicated that NFIX protein is markedly enriched in GBM and may play a role in the progression of GBM.

### NFIX deficiency attenuates malignant progression of GBM in mice

**Fig. 2:**
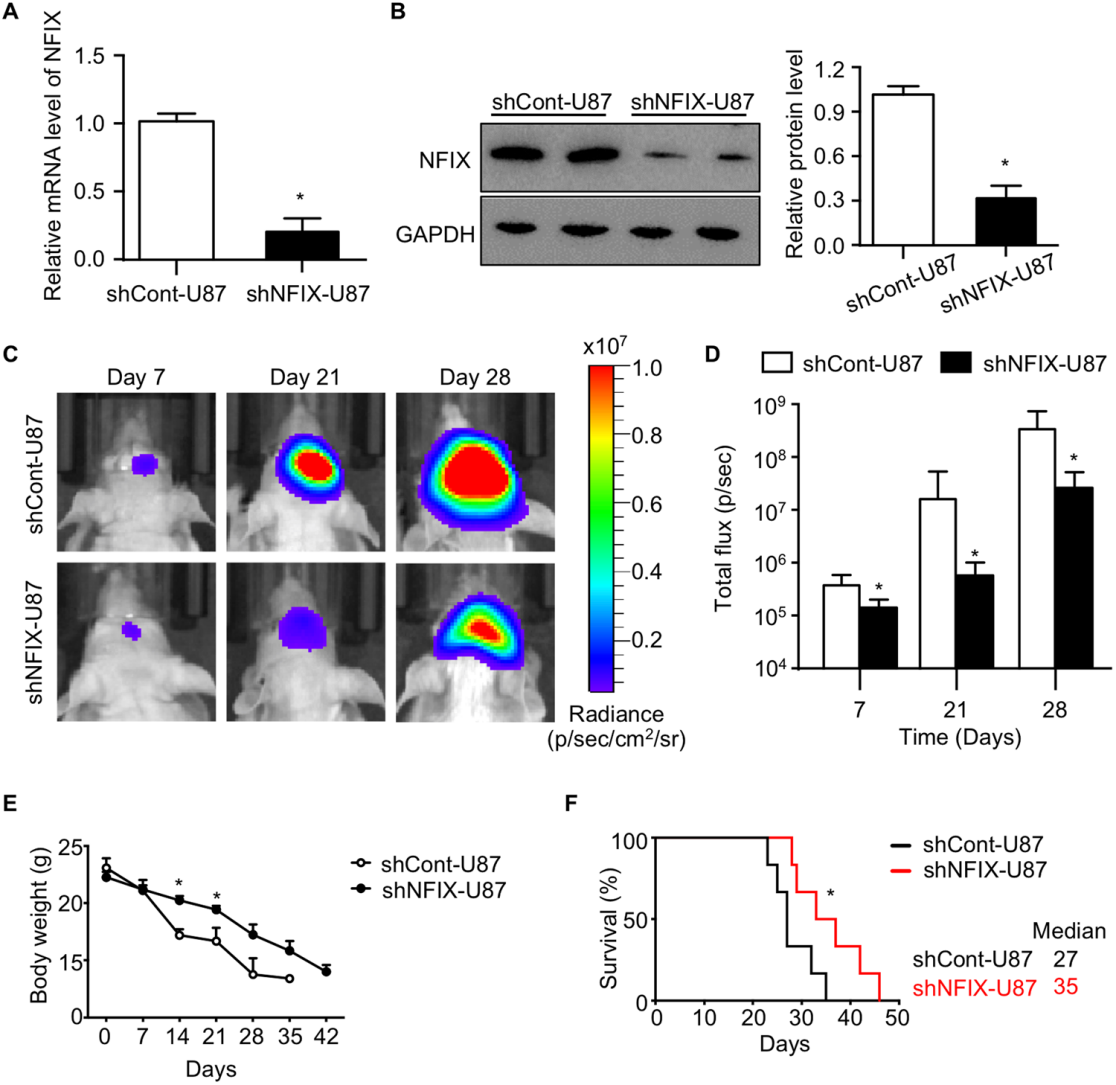
NFIX deficiency attenuates malignant progression of GBM in mice. shNFIX-U87 and shCont-U87 cells were used. **a** Relative mRNA levels of *NFIX* normalized with *GAPDH* in shNFIX-U87 cells (*n* = 6). **b** Immunoblotting analysis of NFIX and GAPDH in shNFIX-U87 cells. Representative images are shown. The bar chart is a relative expression level of NFIX normalized with GAPDH (*n* = 5). **c**–**f** shNFIX-U87 and shCont-U87 cells were implanted orthotopically into the hippocampus of immunodificient nude mice. **c** In vivo bioluminescent imaging of nude mice at day 7, 21, and 28 post implantation (*n* = 6). **d** Quantification of luminescence signal intensity from orthotopic tumor on day 7, 21, and 28 after implanting the indicated GBM cells (*n* = 6). **e** Body weight of nude mice implanted with U87 cells stably expressing shNFIX or control shRNA (*n* = 6). All data are represented as the mean ± s.e.m. *p < 0.05, shCont-U87 vs. shNFIX-U87 group (Student’s *t* test). **f** Survival curve of nude mice implanted with U87 cells stably expressing shNFIX or control shRNA (*n* = 6). Medians are shown. *p < 0.05, shCont-U87 vs. shNFIX-U87 group (log-rank test).

To explore the functional role of NFIX in the progression of GBM, we first generated a U87 human GBM cell line with stable knockdown of NFIX using lentiviral shRNA. Three NFIX specific shRNAs were evaluated in U87 cells. shRNA3 showed greatest knockdown and was selected for all subsequent experiments (shRNA3 was defined as shNFIX; Fig. S1a, b). The protein level of NFIX was reduced by >60% upon shNFIX knockdown, as revealed by QPCR and westernblot analysis (Fig. 2a, b). Next, we orthotopically implanted U87 GBM cells with or without NFIX downregulation into the hippocampus of immunodeficient nude mice. U87 cells transduced with lentiviral shNFIX (shNFIX-U87 cells) suppressed the tumor enlargement in the brain of nude mice as revealed by the in vivo bioluminescent imaging (Fig. 2c, d), suggesting that the malignant progression of GBM in the mice is attenuated by NFIX silencing. Mice implanted orthotopically with shNFIX-U87 cells delayed body weight loss and prolonged lifespan (Fig. 2e, f). Meanwhile, we extracted the protein from orthotopic tumors of nude mice. The protein expression level of NFIX was significantly reduced in mice implanted orthotopically with shNFIX-U87 cells (Fig. S2a, b), further confirming the NFIX silencing in vivo. Taken together, these results demonstrated that NFIX deficiency attenuates the malignant progression of GBM in mice.

### Knockdown of NFIX impairs proliferation and migration of GBM cells

**Fig. 3:**
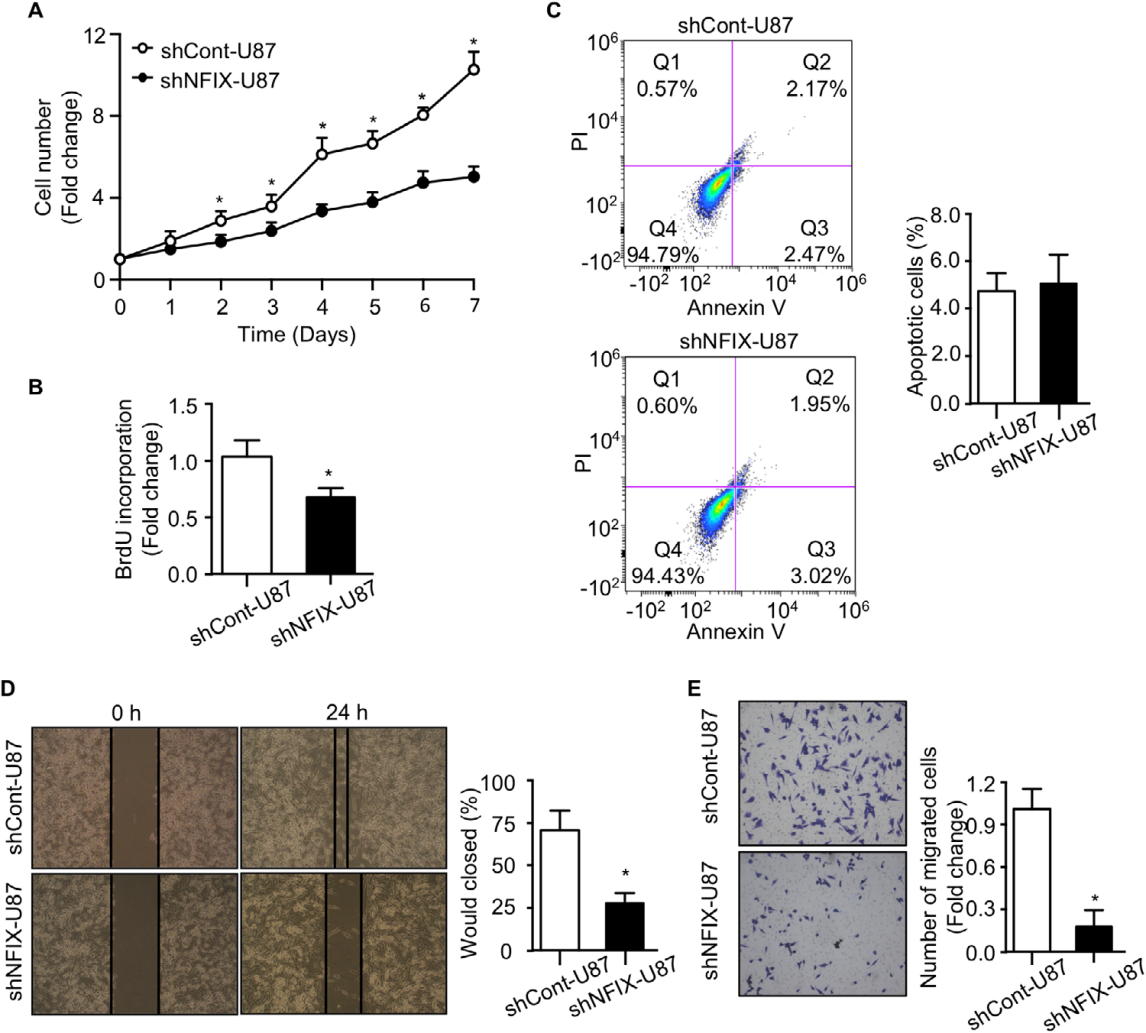
Knockdown of NFIX impairs proliferation and migration of GBM cells. shNFIX-U87 and shCont-U87 cells were used. **a** Cell number was determined by trypan blue assay at indicated time points (*n* = 6). **b** BrdU incorporation of cells (*n* = 6). **c** Cells were stained with Annexin V and propidium iodide (PI), followed by flow cytometry analysis. The right panel is the percentage of apoptotic cells (*n* = 6). **d** Wound healing assay of cells was determined at 0 and 24 h after wound was created. The right panel is the percentage of would closed at 24 h (*n* = 6). **e** Transwell assays of U87 cells stably expressing shNFIX or control shRNA. The right panel is the quantification of the number of migrated cells (*n* = 6). All data are represented as the mean ± s.e.m. *p < 0.05, shCont-U87 vs. shNFIX-U87 group (Student’s *t* test).

To probe the underlying mechanism of NFIX in the regulation of GBM progression, shNFIX-U87 cells and shCont-U87 cells were subjected to the assessment of proliferation, viability and migration. Knockdown of NFIX inhibited the GBM cell proliferation, as reflected by trypan blue and BruD incorporation assays (Fig. 3a, b). However, the apoptosis of U87 cells was not affected by the NFIX knockdown (Fig. 3c). On the other hand, cell migration capacity of U87 cells transduced with either lentiviral shNFIX or control shRNA was determined using wound healing and transwell assays. The wound healing assay showed that shNFIX-U87 cells closed the wound more slowly than shCont-U87 control cells (Fig. 3d). In addition, the inhibitory effect of NFIX deficiency on cell migration was also observed when we employed transwell assay (Fig. 3e). Consistently, impaired proliferation and migration were shown in another established GBM cell line U251 (Fig. S3a–d). Collectively, these findings suggested that NFIX plays a role in the regulation of proliferation and migration but not viability of GBM cells.

### NFIX deficiency downregulates Ezrin expression in GBM cells

**Fig. 4:**
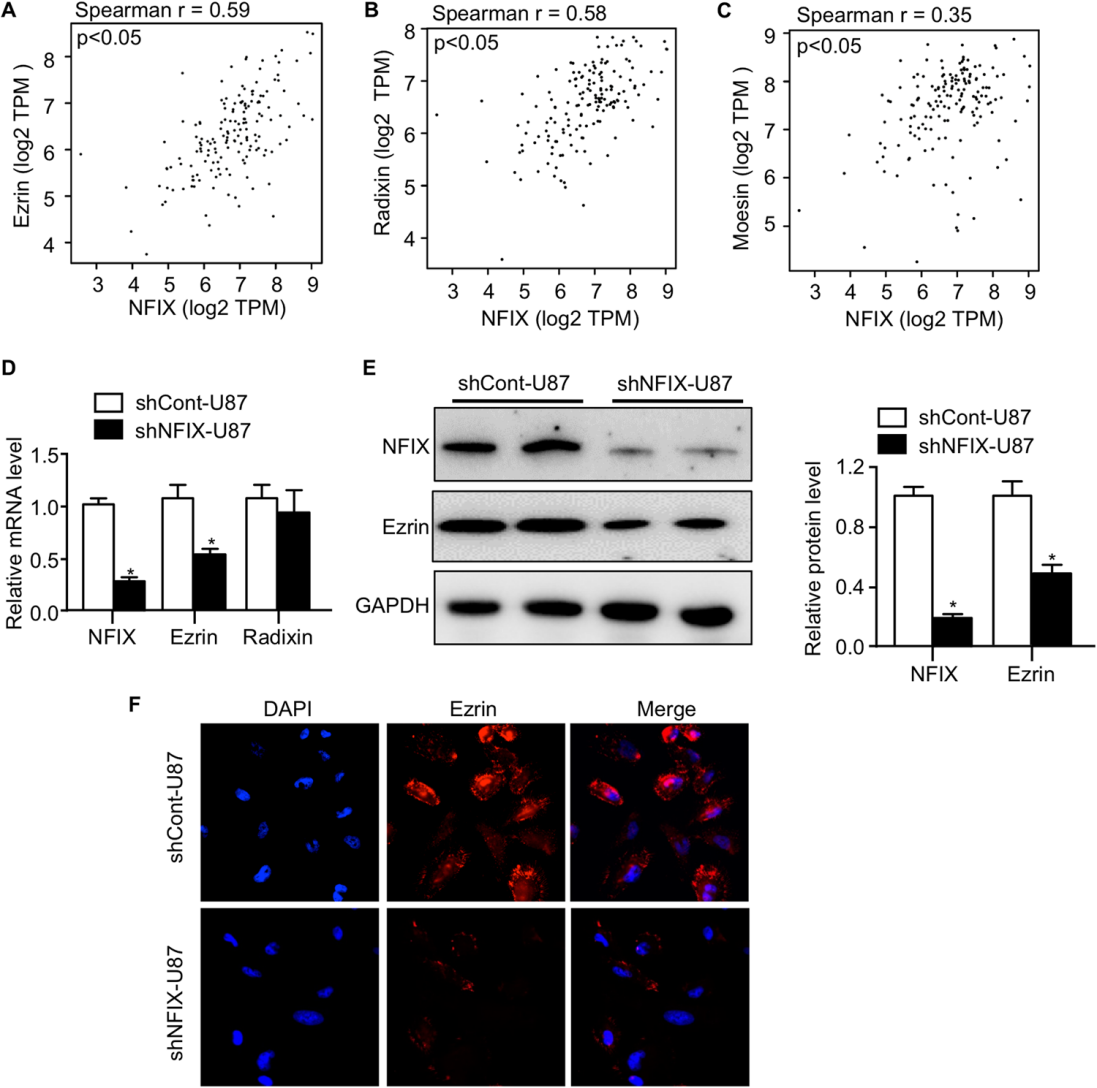
Knockdown of NFIX downregulates Ezrin expression in GBM cells. **a**–**c** Correlation analysis of *NFIX* mRNA level with **a** *Ezrin*, **b** *Radixin*, and **c** *Moesin* in human GBM samples (*n* = 163). Spearman’s test. **d** Relative mRNA levels of *NFIX*, *Ezrin*, and *Radixin* normalized with *GAPDH* in shNFIX-U87 and shCont-U87 cells (*n* = 6). **e** Immunoblotting analysis of NFIX, Ezrin, and GAPDH in shNFIX-U87 and shCont-U87 cells. Representative images are shown. The bar chart is a relative expression levels of NFIX and Ezrin normalized with GAPDH (*n* = 5). **f** Immunofluorescent staining for Ezrin and DAPI in shNFIX-U87 and shCont-U87 cells. Representative images are shown. All data are represented as the mean ± s.e.m. *p < 0.05, shCont-U87 vs. shNFIX-U87 group (Student’s *t* test).

Next, we aimed to explore how NFIX modulates the in vivo growth and migration of GBM cells. Ezrin-Radixin-Moesin (ERM) family, which crosslinks actin cytoskeleton and plasma membrane, plays an emerging role in cell migration^27,28^. To investigate whether there is an association between NFIX and ERM family, we performed correlative analysis in the 163 GBM human subjects via the Gene Expression Profile Interactive Analysis^29^. Interestingly, the *Ezrin* and *Radixin* but not *Moesin* mRNA expression were strongly and positively correlated with *NFIX* (Fig. 4a–c), suggesting that NFIX may regulate the migration of GBM cells in the Ezrin- or Radixin-dependent manner. However, knockdown of NFIX reduced mRNA abundance of *Ezrin* decreased but had no effect on *Radixin* in U87 cells (Fig. 4d). Consistently, protein level of Ezrin was also decreased followed with NFIX knockdown in U87 GBM cells (Fig. 4e). Immunofluorescent staining further supported that NFIX silencing downregulated Ezrin expression in GBM cells (Fig. 4f). These findings suggested that NFIX may promote malignant growth and migration of GBM cells via the induction of Ezrin.

To further determine the role of Ezrin in the regulation of GBM cell migration, we employed siRNA against Ezrin (siEzrin) to downregulate the expression of Ezrin in human U87 GBM cell line. mRNA and protein levels of Ezrin were effectively downregulated by siEzrin as revealed by QPCR and immunoblotting analysis (Fig. S4a, b). U87 GBM cells with Ezrin silencing displayed decreased invasion and migration (Fig. S4c, d), which is consistent with previous study^27^. Consistently, impaired migration was also observed in U251 GBM cells with Ezrin knockdown (Fig. S5a, b). These findings suggest that Ezrin is an essential regulator for invasion and migration of GBM cells. Since NFIX and Ezrin display strong correlation in GBM, we hypothesized that NFIX promotes the migration and invasion of GBM cells through its transcriptional activation of Ezrin.

### Ezrin rescues defective migration in GBM cells with NFIX deficiency

**Fig. 5:**
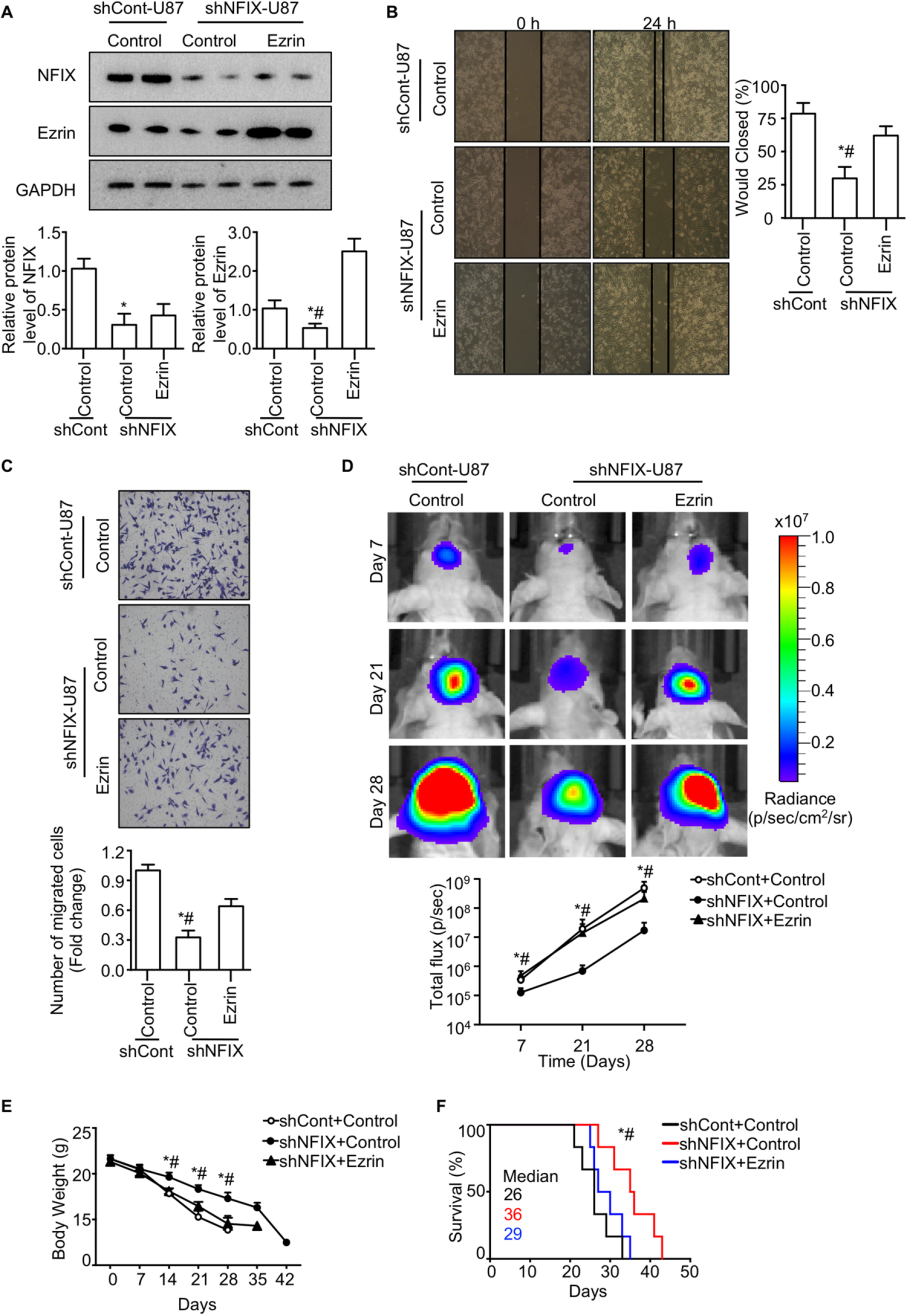
Ezrin rescues defective migration in NFIX-null GBM cells. shNFIX-U87 and shCont-U87 cells overexpressing Ezrin were generated by lentivirus-mediated overexpression and were subjected to the following experiments. **a** Immunoblotting analysis of NFIX, Ezrin, and GAPDH in cells. Representative images are shown. The bar chart is a relative expression level of NFIX and Ezrin normalized with GAPDH (*n* = 6). **b** Wound healing assay (*n* = 6). **c** Transwell assays (*n* = 6). **d**–**f** Indicated U87 cells were implanted orthotopically into the hippocampus of immunodificient nude mice. **d** In vivo bioluminescent imaging of nude mice at day 7, 21, and 28 post implantation. The bar chart is a luminescence signal intensity from an orthotopic tumor on day 7, 21, and 28 after implanting the indicated GBM cells (*n* = 6). **e** Body weight (*n* = 6). All data are represented as the mean ± s.e.m. *p < 0.05, shNFIX+Control vs. shCont+Control; ^#^*p* < 0.05, shNFIX+Control vs. shNFIX+Ezrin (one-way ANOVA). **f** Survival curve (*n* = 6). Median are shown. *p < 0.05, shNFIX+Control vs. shCont+Control; ^#^*p* < 0.05, shNFIX+Control vs. shNFIX+Ezrin (log-rank test).

To examine whether Ezrin is the downstream mediator of NFIX, we replenished Ezrin to shNFIX-U87 cells using a lentiviral-mediated overexpression system (Fig. 5a). Results from wound healing and transwell assay illustrated that impaired migration of shNFIX-U87 cells was largely reversed by ectopically overexpression of Ezrin (Fig. 5b, c). Replenishment of Ezrin also largely rescued the impaired migration of U251 cells with NFIX silencing (Fig. S6a, b). In addition, shNFIX-U87 cells with replenishment of Ezrin promoted the tumor malignant growth in brain of nude mice when compared with the control group (Fig. 5d). NFIX-null effects on the body weight and lifespan were not observed in the mice implanted orthotopically with shNFIX-U87 cells replenished with Ezrin (Fig. 5e, f). Furthermore, similar patterns of the protein levels of NFIX and Ezrin in orthotopic tumor were observed compared with that of in vitro U87 cells (Fig. S7a–c). Taken together, these results showed that expression of Ezrin can rescue defective migration of the GBM cells in the absence of NFIX and further corroborated Ezrin as a downstream target of NFIX.

### Identification of NFIX binding site in the promoter of Ezrin

**Fig. 6:**
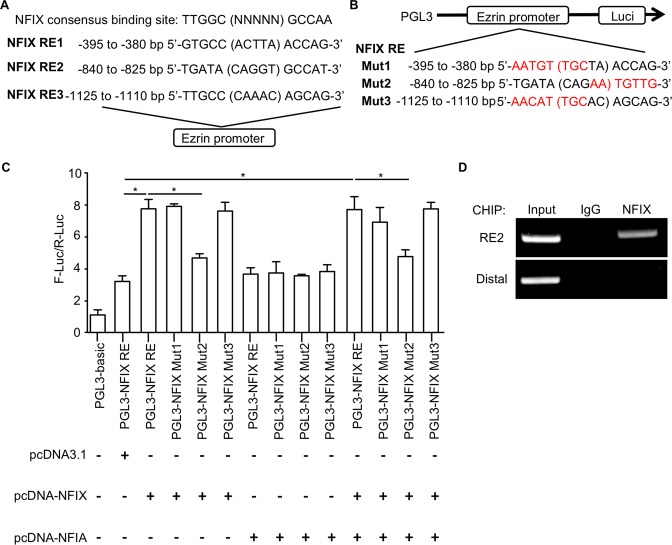
Identification of the NFIX binding site in *Ezrin*. **a** Location and sequences of NFIX putative response elements (REs) identified in the promoter region of *Ezrin* gene. **b** PGL3 reporter plasmids encode luciferase under the control of human *Ezrin* gene promoter. The putative NFIX REs within the promoter region were mutated as Mut1, 2, and 3. **c** Measurement of firefly-luciferase activity normalized with the renilla-luciferase activity (*n* = 6). **d** U87 cells were subjected to chromatin immunoprecipitation using anti-NFIX or anti-IgG antibody, and followed with PCR amplification using primers specific to NFIX RE2 of *Ezrin* promoter region or distal region of *Ezrin* as negative control. All data are represented as the mean ± s.e.m. *p < 0.05 (Student’s *t* test).

We next sought to identify whether there is potential NFIX binding sites in Ezrin. Using in silico analysis, we identified three putative NFIX response elements (REs) in the promoter region of *Ezrin* gene (Fig. 6a). To investigate whether NFIX regulates the promoter activity of *Ezrin* via the putative NFIX REs, we cloned the promoter region containing the NFIX REs of the *Ezrin* gene into the firefly-luciferase reporter vector pGL3 (Fig. 6b). Results from dual luciferase reporter assay showed that both single expression of NFIX and combined expression of NFIA and NFIX significantly enhanced the promoter activity of *Ezrin* in GBM cells when compared with those vector control (Fig. 6c). However, NFIA single expression had no effect on *Ezrin* transcriptional activity. These findings suggested the specificity of NFIX on the transcriptional regulation of *Ezrin* (Fig. 6c). To further identify the specific NIFX REs, we generated three constructs with NFIX REs mutation (Fig. 6b). The induction of NFIX on *Ezrin* promoter activity was lost in mutation of NFIX RE2 (Fig. 6c). Furthermore, chromatin immunoprecipitation analysis illustrated that there was an enrichment of NFIX to the NFIX RE2 in the *Ezrin* promoter (Fig. 6d), suggesting that NFIX can directly bind to the Ezrin promoter. Together, these findings suggested that NFIX induces *Ezrin* expression by directly interacting with NFIX RE2 in the promoter region, triggering the transcriptional induction of Ezrin.

## Discussion

Induced migration and invasion of GBM cells are two hallmarks of malignant progression of GBM. However, the underlying mechanisms remain largely unknown. In this study, we showed that the NFIX-Ezrin axis plays an important role in regulating the migration of GBM cells. In addition, we demonstrated that NFIX, as a transcription factor, regulates Ezrin expression by directly binds to the promoter region (−840 to −825 bp) of *Ezrin* gene. Our study, for the first time, showed that NFIX is dramatically upregulated in GBM and plays a tumor-promoting role in GBM development.

NFIX has been shown to be involved in the progression of esophageal squamous cell carcinoma and colorectal cancer^22,23^. Opposite to the tumor-promoting role in the GBM, NFIX inhibits the migration of cancer cells in both esophageal squamous cell carcinoma and colorectal cancer. These findings indicate that NFIX plays distinct roles in different types of cancer, thus the employment of inhibitors against NFIX for the cancer therapy requires further investigation. In addition, these two studies have not explored the underlying mechanism of how NFIX modulates the migration of cancer cells. Using in vitro and in vivo approaches, our study demonstrated that NFIX promotes migration of GBM cells via the upregulation of Ezrin expression.

Ezrin is a member of ERM family, which modulates multiple signaling pathways implicated in cell adhesion and migration^28^. Emerging evidences shows that Ezrin is crucial for migration during the progression of different types of cancer^27,30–33^. On the one hand, Ezrin promotes cancer cell migration mainly through the disruption of cell–cell contact^28^. It has been suggested that Ezrin can recruit and activate Fes kinase, which contributes to disassembly of cell–cell contact^34,35^. On the other hand, Ezrin can be activated by protein kinase C, resulting in CD44-dependent directional migration^36^. Protein level modification of Ezrin was observed in malignant progression of GBM. For example, phosphorylation of Ezrin was demonstrated to be essential for the cancer cell migration and the extent of cancer malignancy, thus previous studies mainly focused on unlocking the mechanisms for phosphorylation regulation of Ezrin protein and the upstream regulators mastering Ezrin phosphorylation^37^. However, our findings uncovered a novel upstream regulation model that NFIX functions as a transcriptional regulator of Ezrin in GBM and promotes Ezrin expression through transcriptional activation.

Role of NFIX on proliferation has been reported in neural progenitor cells and granule neuron precursor cells^38,39^. Our findings also showed that NFIX can promote proliferation in GBM cells (Fig. 3a, b). However, the underlying mechanism of how NFIX modulates proliferation in GBM cells is still unclear. Lee et al. have demonstrated that NFIA accelerates proliferation in GBM cells through the negative regulation of p21, a key mediator for cellular senescence^13^. Although this study implied that proliferation-promoting role of NFIX may be mediated via p21, further exploration on the underlying mechanism of how NFIX modulates proliferation in GBM cells is required.

Although four member of NFI family (NFIA, NFIB, NFIC, and NFIX) share the consensus binding site, several studies have already showed that four NFI members in vitro displayed distinguished preference to same genes^10,40,41^. For instance, single NFIB, NFIC or NFIX, but not NFIA was sufficient to regulate HEY1 transcriptional activity^10^. In the regulation of WAP gene transcription, NFIB and NFIA exerted opposite influence. NFIB preferentially promoted WAP gene transcription, while NFIA suppressed WAP gene transcription^40^. In this regard, it is interesting to explore the underlying mechanism on differential regulation between NFIA and NFIX on Ezrin gene transcription in the future.

In summary, this is the first study uncovering that NFIX can accelerate GBM development by enhancing the migration capacity of GBM cells in an Ezrin-dependent manner. NFIX directly interact with the promoter region of *Ezrin*, inducing the Ezrin expression in transcriptional level. Our findings provide novel insights into the role of NFIX in malignant GBM development. Nowaday, siRNA has become a powerful drug, which has been recently used in therapeutic applications^42^. On August 10, 2018, the first siRNA-based therapeutic drug Onpattro was approved by FDA, for the treatment of Hereditary Transthyretin Amyloidosis. As for cancer therapy, several RNAi drugs are under clinical trial stages^42^. Since there are many potential candidates currently at phase 3 clinical trials, we believe that more RNAi-based drugs will be aprroved by FDA in the coming years. Our study indicates that siRNA targeting NFIX can be a new therapeutic treatment for GBM.

## Materials and methods

### Animal studies

All the NOD/SCID nude mice are on the BALB/cJ genetic background. The 6-week-old male nude mice were obtained from Gempharmatech Company and were randomly divided into three groups (6 mice per group). The investigators were not blinded to the experimental groups. The mice were maintained under filter air barrier conditions and had free access to sterilized water and standard chow and were housed in a room with 23 °C temperature and 12 h light/dark cycle control. Orthotopic implantation of GBM cells into the hippocampus of nude mice was performed as previous described (*n* = 6)^13,43^. Anesthetized nude mice were fixed in the stereotaxic frame (catalog #JTND-1S; Beijin Getimes Technology). A small hole was made into the skull 1.7 mm lateral and 0.5 mm anterior to the bregma using a drill. 200,000 firefly-luciferase labeled GBM cells in 2 μL phosphate-buffered saline (PBS) were injected into the hole to a depth of 3.2 mm through a micro-injection needle (catalog #88011; Hamliton) at a rate of 1 μL/minute by micro-injection system (catalog #TYD01-01; Lead Fluid). The needle was required to retain in place for 5 min before the withdrawing the needle. Body weight was measured weekly. Intracranial tumor size was monitored by IVIS Spectrum in vivo imaging system (PerkinElmer) 10 min after intraperitoneal injection of luciferin (catalog #P1043; Promega). All animal protocols were approved by the Animal Experimentation Ethics Committee, Southern Medical University.

### Real-time quantitative PCR

Total RNA was extracted using TRIzol (catalog #15596018; Thermo Fisher Scientific) and the cDNA was generated using the reverse-transcription kit (catalog #A5000; Promega). Real-time quantitative PCR was performed in Real-time PCR System (Applied Biosystems 7500) using SYBR Green (catalog #AQ131-02; Trans) with the gene-specific primers (Table S1).

### Lentiviral vector production and transduction

Lentiviral-shRNA vector (shNFIX; Target sequence-ACTGGATCTTTATCTGGCTTA), lentiviral-overexpression vectors (Ezrin) and their negative control vectors were generated by Gene Chem. To package the lentivirus, the vectors were transfected into 293T cells line with the psPAX2 and pMD2.g using lipofectamine 3000 reagent (catalog #L3000015; Thermo Fisher Scientific) and Opi-MEM (catalog #L31985070; Thermo Fisher Scientific). Lentivirus-containing supernatant were collected at 48 and 72 h after transfection, followed by 0.45 μm filter and ultracentrifugation. Human primary GBM cell lines U87 and U251 were used for lentiviral transduction. All cell lines were mycoplasma-free and have been authenticated using short tandem repeat profiling with 6 months (reports were shown in Supplementary materials). To generate shNFIX-U87/U251 cells, U87/U251 cells were transduced with shNFIX lentiviral supernatant in the presence of 3 μg/mL polybrene (catalog #TR1003; Sigma). Forty-eight hours after transduction, the cells were subjected to antibiotic selection using puromycin (catalog #A1113803; Thermo Fisher Scientific) for 12 days. To further generate shNFIX-U87/U251 cells with Ezrin replenishment, shNFIX-U87/U251 cells were transduced with Ezrin lentiviral-overexpression supernatant in the presence of 3 μg/mL polybrene, followed by hygromycin (catalog #10687010; Thermo Fisher Scientific) selection for 12 days.

### siRNA knockdown

siRNA against Ezrin and control siRNA were generated by Sango Biotech (Table S2). U87 or U251 cells were seeded in DMEM with 10% FBS. U87 or U251 cells were transfected when the cells were 70% confluent. Cells were transfected with siRNA using Opi-MEM and lipofectamine 3000 reagent according to the manufacturer’s instructions.

### Immunoblotting

Proteins from tissues or cells were prepared with a RIPA lysis buffer (catalog #9803; Thermo Fisher Scientific) with phosphatase inhibitor and protease inhibitor cocktail (catalog #11697498001; Sigma). Proteins were electrophoresed by SDS-PAGE and were transferred onto polyvinylidene difluoride membranes. Membranes were immunostained with primary antibodies against NFIX (catalog #ab101341; Abcam), Ezrin (catalog #3145S; Cell Signaling Technology) or GAPDH (catalog #2118; Cell Signaling Technology), followed by incubation with horseradish peroxidase-conjugated secondary antibody (catalog #7074S; Cell Signaling Technology). The protein bands were visualized by imaging system (Bio-Rad ChemiDoc^TM^ Imaging System) and quantified using ImageJ software. Full images of immunoblotting were shown in Figure S8.

### Tissue microarray (TMA) and immunohistochemical (IHC) staining

Clinical human glioma lesions and normal brain tissue (obtained from surgical trauma patients; *n* = 8 for normal brain tissue, *n* = 25 for WHO II, *n* = 26 for WHO III and *n* = 19 for GBM) were collected from 2017 to 2019 in Yijishan Hospital and Shenzhen Hospital of Southern Medical University under the surveillance of their Human Ethics Committees. Samples from patients who received preoperative radiation or chemotherapy were excluded. Informed consent was obtained from all subjects. Each sample was confirmed by H&E staining and pathologist. A TMA that contains normal brain tissue, glioma cancer lesions with different WHO stages was constructed. TMA sections were deparaffined with xylene and washed in serial dilutions of ethanol. Tissue sections were subjected to antigen retrieval by boiling in a sodium citrate buffer (10 mmol/L, pH 4.5). After blocking using PBS with 10% FBS and 3% bovine serum albumin (BSA) for an hour at room temperature, tissue sections were incubated with anti-NFIX antibody (1:600; catalog #ab101341; Abcam) overnight at 4 °C. Next day, sections were incubated with horseradish peroxidase and Diaminobenzidine detection kit (catalog #PV-6000; ZSGB-BIO). Finally, tissue sections were counterstained for nuclei with hematoxylin solution and subjected to microscopy analysis. The staining results were assessed by two independent investigators blinded to patients’ information.

### Cell count assay

Cells were seeded at a density of 2 × 10^4^ cells per well in 12-well plates containing DMEM with 10% FBS and 1% penicillin-streptomycin. Cells were removed by trypsinization every 24 h and the number of viable cells was determined by trypan blue staining (*n* = 6).

### BrdU incorporation assay

Cell proliferation was measured using BrdU cell proliferation assay kit (catalog #6813; Cell Signaling Technology) according to manufacturer’s instruments (*n* = 6). Absorbance was determined at 450 nm using Synergy H1 microplate reader (BioTek). The experiment was repeated three times.

### Apoptosis assay

Cells stably expressing shNFIX or control shRNA were stained with Annexin V and PI (catalog #C1062S, Beyotime) for 15 min at room temperature protecting from light. The stained cells were subjected to flow cytometry analysis using Sony SA3800 analyzer (*n* = 6). The percentage of apoptotic cells was calculated by annexin V positive (Q3) plus Annexin V and PI double positive (Q2). The experiment was repeated three times.

### Immunofluorescent staining

Cells were seeded in 12-well plate and were cultured for 24 h. Cells were fixed with 4% paraformaldehyde in PBS for 10 min at room temperature and washed with PBS for three times. To permeabilize the membrane, cells were incubated with 0.1% Triton X-100 (catalog #HFH10; Thermo Fisher Scientific) in PBS for 15 min at room temperature. Next, PBS with 10% FBS and 3% bovine serum albumin (BSA) was used to block the cells for an hour at room temperature. After blocking, cells were incubated with the anti-Ezrin antibody (1:200; catalog #3145 S; Cell Signaling Technology) overnight at 4 °C. After washing three times with PBS, the cells were incubated with a red fluorescent anti-rabbit IgG (1:500; catalog #A11037; Thermo Fisher Scientific) for an hour and followed by DAPI (catalog #P36931; Thermo Fisher Scientific) staining for 10 min at room temperature.

### Wound healing assay

Cells were seeded in 12-well plate and were cultured in DMEM (catalog #Fl101-01; Trans) with 10% FBS (catalog #10270; Thermo Fisher Scientific) and 1% penicillin-streptomycin (catalog #FG101-01; Trans) until the formation of monolayer. A scratch was made in the center of each wells using a P20 pipette tips. Images of cells were recorded at 0 h and 24 h post scratching. The area of opening space at 24 h was shown relative to the time point 0 h. The area of opening space at 0 h were defined as 100% (*n* = 6).

### Transwell migration assay

Cells were seeded in the top chambers of 24-well cell culture Transwell inserts (catalog #CLS3464; Corning) containing DMEM with 10% FBS and 1% penicillin-streptomycin. Cells were allowed to migrate through the 8.0 μm polyethylene terephthalate membrane toward the lower chambers containing DMEM with 10% FBS and 1% penicillin-streptomycin. After 24 h, cells were fixed with 100% methanol for 15 min, stained with 1% crystal violet (catalog #C6158; Sigma) in 20% methanol for 15 min at room temperature and followed by microscopy analysis (*n* = 6).

### Luciferase reporter gene assay

U87 GBM cells were cultured in 12-well cell culture plates and followed by transfection with indicated vectors using lipofectamine 3000 (catalog #L3000-01; Thermo Fisher Scientific) for 24 h. Double-luciferase activity was determined by luciferase reporter assay kit (catalog #FR201; Trans) according to the manual. In brief, cells were harvested using cell lysis buffer for 10 min at room temperature. Cell lysate was centrifuged at 12,000 rpm for 10 min at 4 °C. 20 μL of supernatant was added into 100 μL reaction buffer with firefly substrate, followed with the measurement of firefly-luciferase activity using Synergy H1 microplate reader (BioTek). Next, 100 μL reaction buffer with renilla substrate was added into the mixture and followed with the measurement of renilla-luciferase activity. Firefly-luciferase activity was normalized with the renilla-luciferase activity (*n* = 6).

### Chromatin immunoprecipitation

Chromatin immunoprecipitation was performed using Chromatin IP Kit (catalog #9002S; Cell Signaling Technology) according to the manual. U87 cells were cross-linked with 1% formaldehyde for 15 min at room temperature, followed by addition of glycine to 0.125 M to stop the cross-linking reaction. The cell lysate was subjected to sonication to generate DNA fragments. Cell lysates were incubated with an anti-NFIX antibody (catalog #NBP2-15039; Novus) or IgG control antibody (catalog #ab2410; Abcam), followed by incubation with protein G agarose beads (catalog #9007S; Cell Signaling Technology). The complex was eluted by elution buffer, followed with cross-link reversion by incubating the complex at 65 °C for 2 h. DNA was purified using DNA purification columns (catalog #9002S; Cell Signaling Technology). The purified DNA fragments were amplified by PCR using primers specific to NFIX RE2 of *Ezrin* or the 8-kb upstream distal region of *Ezrin* as negative control (Table S1).

### Statistics

Sample size was determined based on previous publication and the variability observed in preliminary experiments. All data are expressed as mean ± s.e.m. All statistical analysis was performed using SPSS or GraphPad Prism 7.0. Equality of variance was assessed by Levene test. The statistical significance was calculated using unpaired Student *t* test (for two groups), one-way ANOVA with Bonferroni correction for multiple comparisons (for three groups), or lon-rank test (for survival curve). Pair-wise gene expression correlation analysis was performed using Spearman’s test. A *p* value of <0.05 represents a significant difference in all statistical comparisons.

## Supplementary information

Supplementary Materials
