# Supplementary material for: Nuclear factor IX promotes glioblastoma development through transcriptional activation of Ezrin
Source: Oncogenesis. 2020 Apr 14;9(4):39. doi: 10.1038/s41389-020-0223-2 (PMC7156762; doi:10.1038/s41389-020-0223-2)
Supplement: Supplementary file 1 — Supplementary Materials [file 41389_2020_223_MOESM1_ESM.pdf]

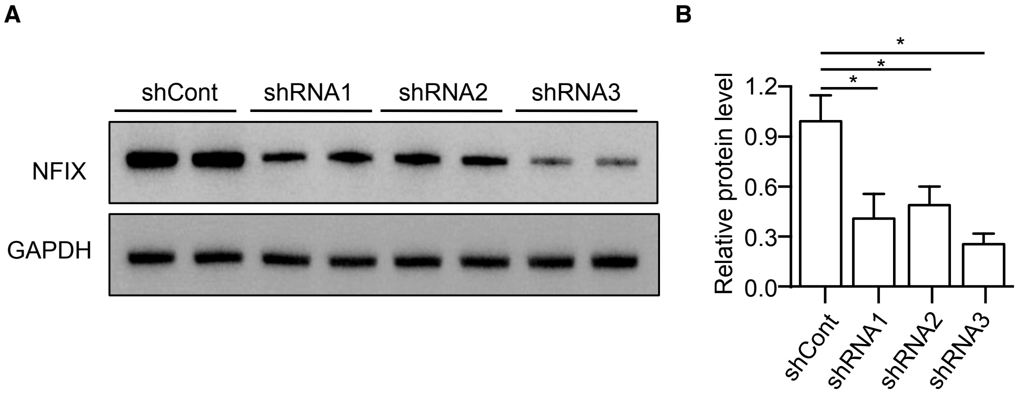

Figure S1. Knockdown of NFIX by shRNAs in U87 cells.

(A) Immunoblotting analysis of NFIX and GAPDH in U87 cells expressing shRNAs targeting human NFIX. Representative images are shown. (B) The bar chart is relative expression level of NFIX normalized with GAPDH (n=6). All data are represented as the mean±s.e.m. *p<0.05 (Student's t test).

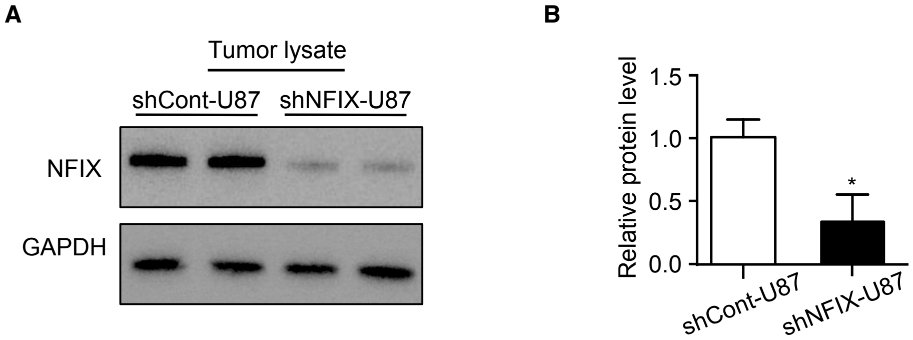

Figure S2. Decreased expression of NFIX in orthotopic tumors.
(A) Immunoblotting analysis of NFIX and GAPDH in orthotopic tumors of nude mice implanted with U87 cells stably expressing shNFIX or control shRNA. Representative images are shown. (B) The bar chart is relative expression level of NFIX normalized with GAPDH (n=6). All data are represented as the mean±s.e.m. *$p<0.05$, shCont-U87 vs. shNFIX-U87 (Student's t test).

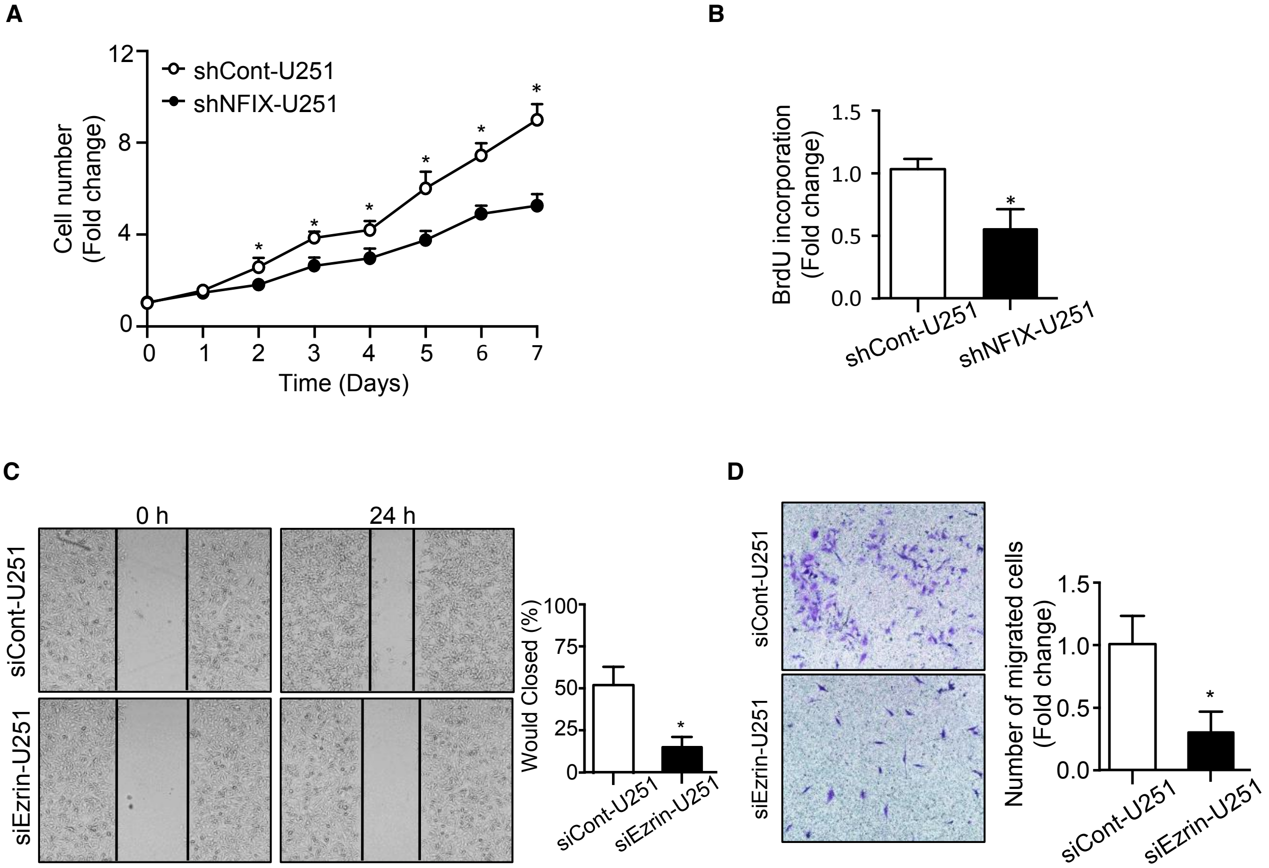

Figure S3. NFIX deficiency inhibits proliferation and migration of U251 GBM cells.
U251 cells stably expressing shNFIX (shNFIX-U251) or control shRNA (shCont-U251) were used. (A) Cell number was determined by trypan blue assay at indicated time points (n=6). (B) BrdU incorporation of cells (n-6). (C) Wound healing assay of cells was determined at 0 and 24 hours after wound was created. The right panel is the percentage of would closed at 24 hours (n=6). (D) Transwell assays of U251 cells stably expressing shNFIX or control shRNA. The right panel is the quantification of the number of migrated cells (n=6). All data are represented as the mean ± s.e.m. *p<0.05, shCont-U251 vs. shNFIX-U251 group (Student's t test).

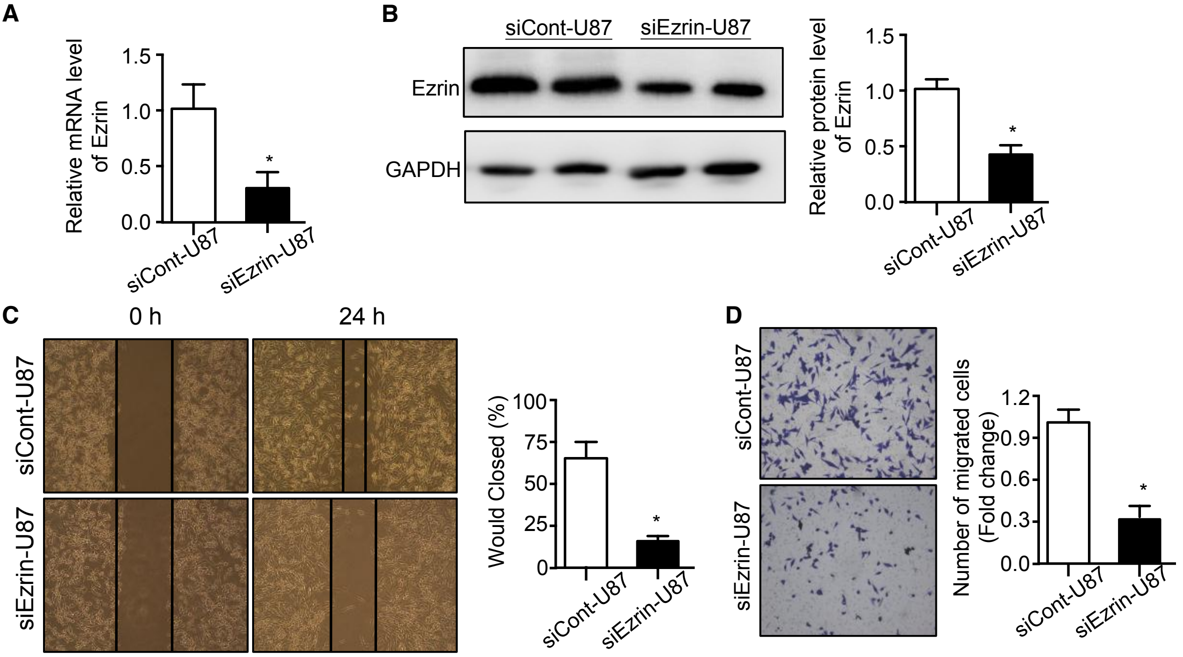

Figure S4. Decreased invasion and migration of U87 GBM cells transfected with siEzrin.
U87 cells were transfected with siRNA against Ezrin (siEzrin-U87 cells) or scramble control (siCont-U87 cells). (A) Relative mRNA levels of *Ezrin* normalized with *GAPDH* in siEzrin-U87 and siCont-U87 cells (n=6). (B) Immunoblotting analysis of Ezrin and GAPDH in siEzrin-U87 and siCont-U87 cells. Representative images are shown. (C) Wound healing assay of cells was determined at 0 and 24 hours after wound was created (n=6). (D) Transwell assays of siEzrin-U87 and siCont-U87 cells (n=6). All data are represented as the mean ± s.e.m. *$p<0.05$, siEzrin-U87 vs. siCont-U87 group (Student's t test).

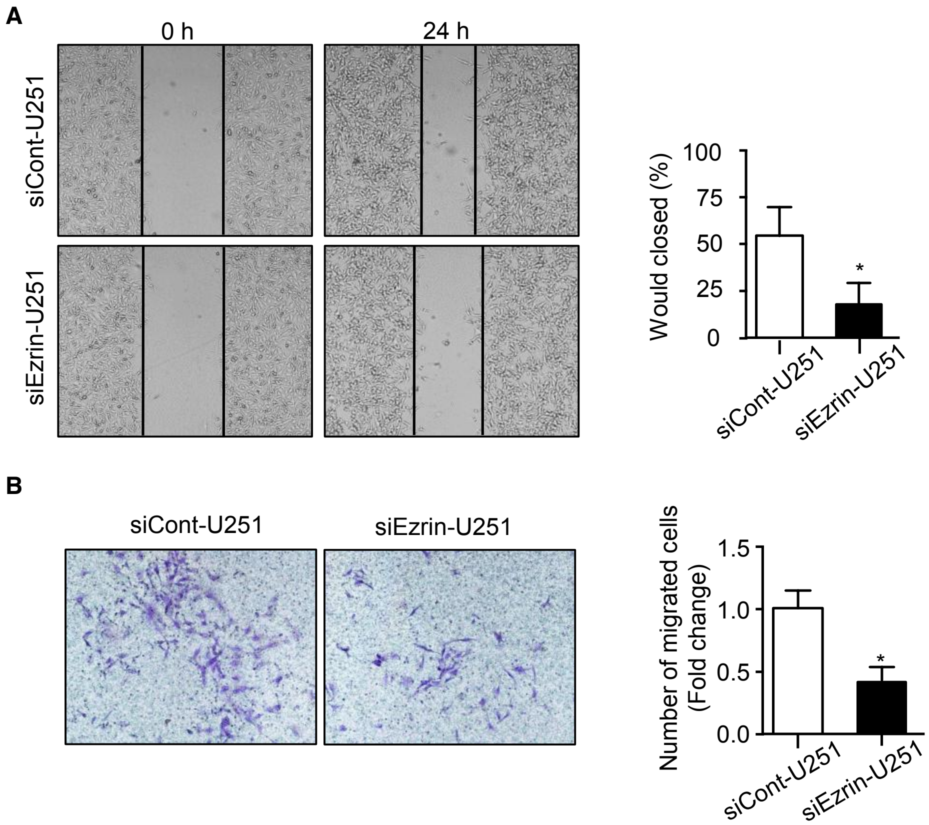

Figure S5. Impaired migration of U251 GBM cells transfected with siEzrin.
U251 cells were transfected with siRNA against Ezrin (siEzrin-U251 cells) or scramble control (siCont-U251 cells). (A) Wound healing assay of cells was determined at 0 and 24 hours after wound was created (n=6). (B) Transwell assays of siEzrin-U251 and siCont-U251 cells (n=6). All data are represented as the mean ± s.e.m. *$p<0.05$, siEzrin-U251 vs. siCont-U251 group (Student's t test).

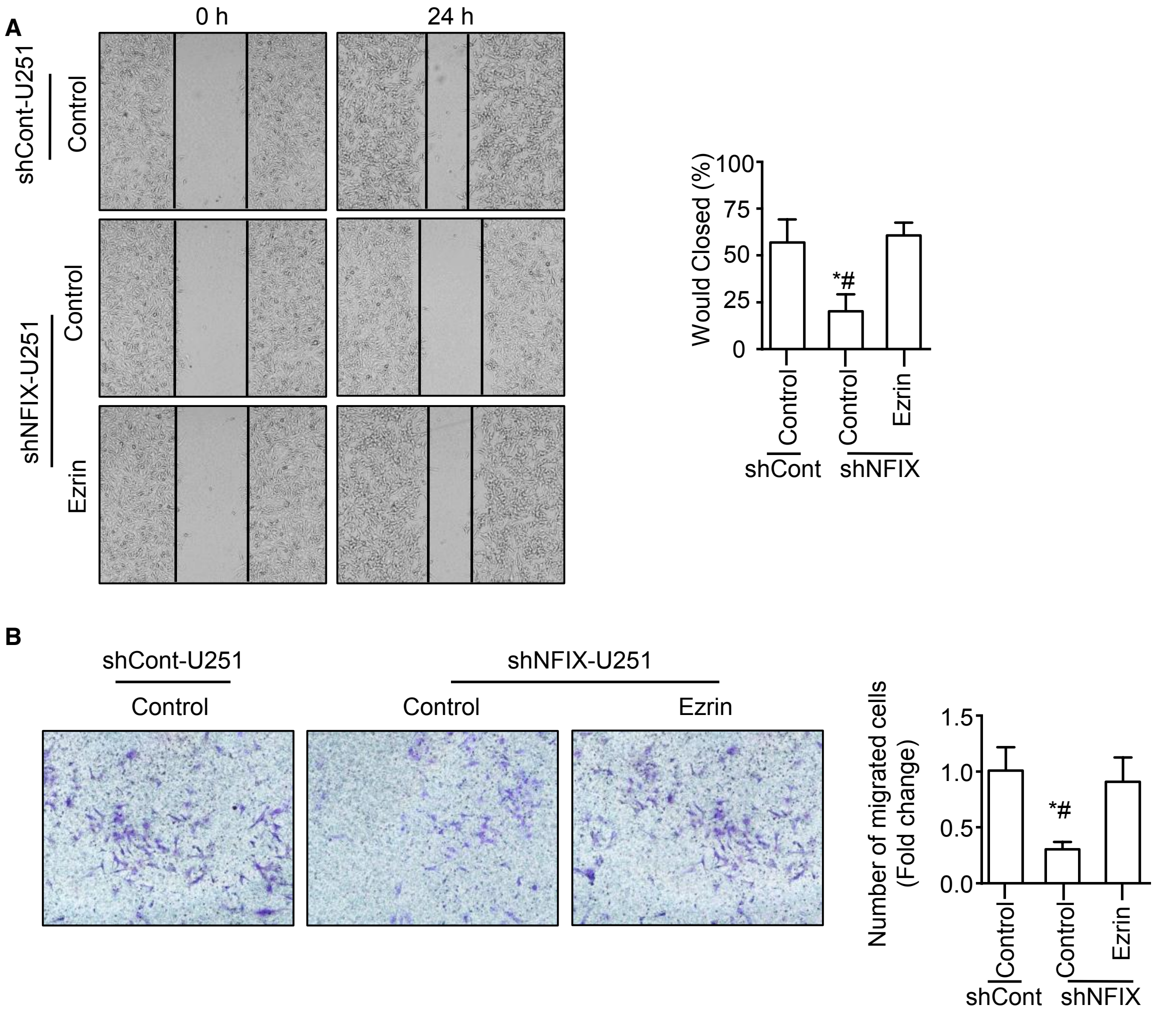

Figure S6. Replenishment of Ezrin reverses defective migration in shNFIX-U251 cells.
shNFIX-U251 and shCont-U251 cells overexpressing Ezrin were generated by lentivirus-mediated overexpression and were subjected to the following experiments. (A) Wound healing assay (n=6). (B) Transwell assays (n=6). *$p<0.05$, shNFIX+Control vs. shCont+Control; #$p<0.05$, shNFIX+Control vs. shNFIX+Ezrin (One-way ANOVA).

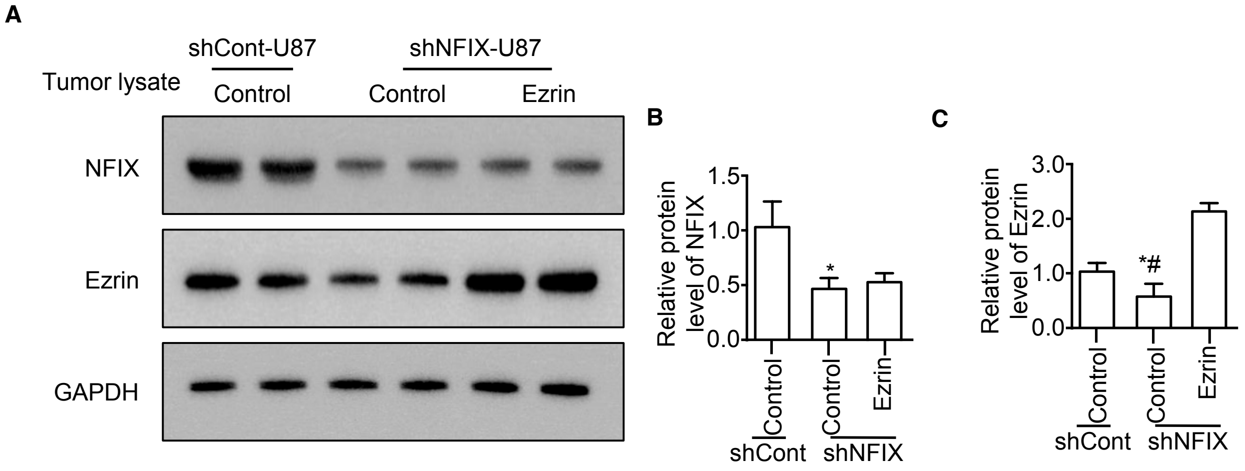

Figure S7. Protein expression levels of NFIX and Ezrin in orthotopic tumors.

(A) Immunoblotting analysis of NFIX, Ezrin and GAPDH in orthotopic tumors of nude mice implanted with indicated U87 cells. Representative images are shown. (B) The bar chart is relative expression level of NFIX normalized with GAPDH (n=6). (C) The bar chart is relative expression level of Ezrin normalized with GAPDH (n=6). All data are represented as the mean±s.e.m. *$p<0.05$, shNFIX+Control vs. shCont+Control; #$p<0.05$, shNFIX+Control vs. shNFIX+Ezrin (One-way ANOVA).

Figure 1

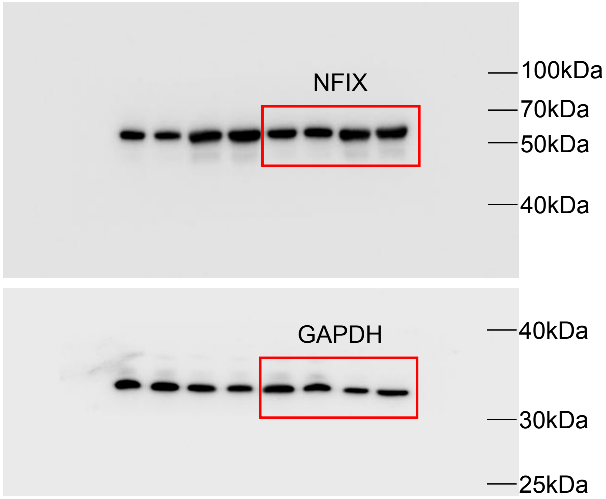

Figure 2

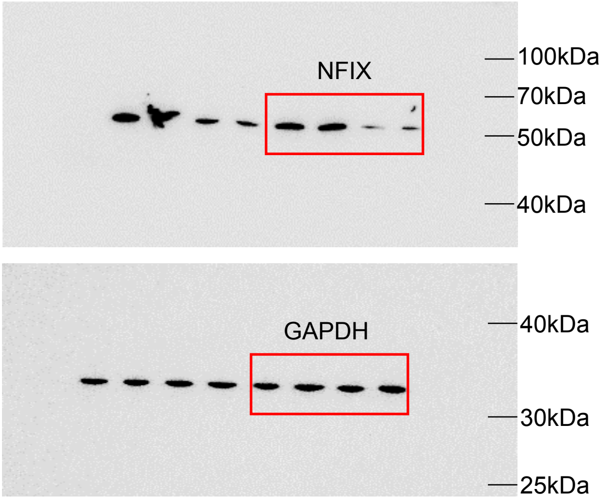

Figure 4

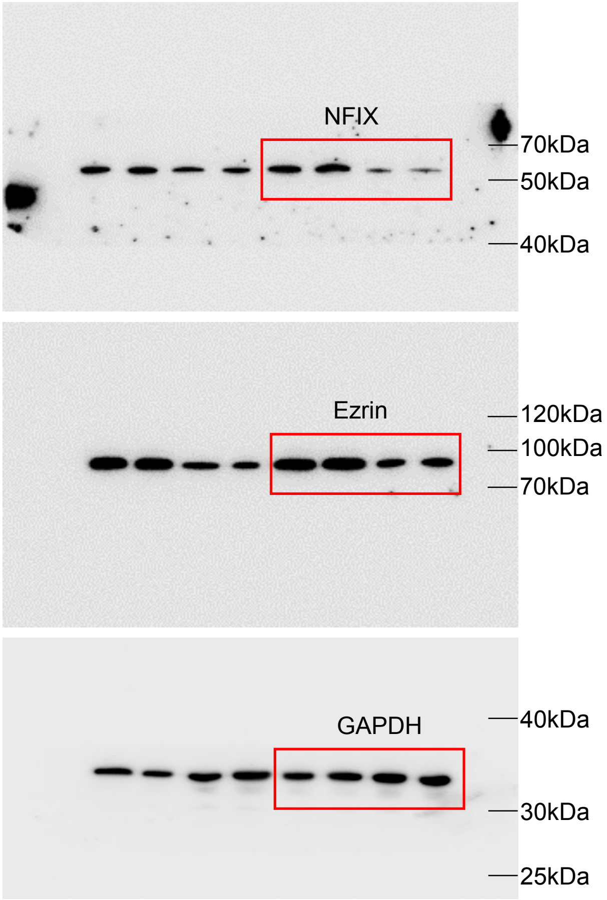

Figure 5

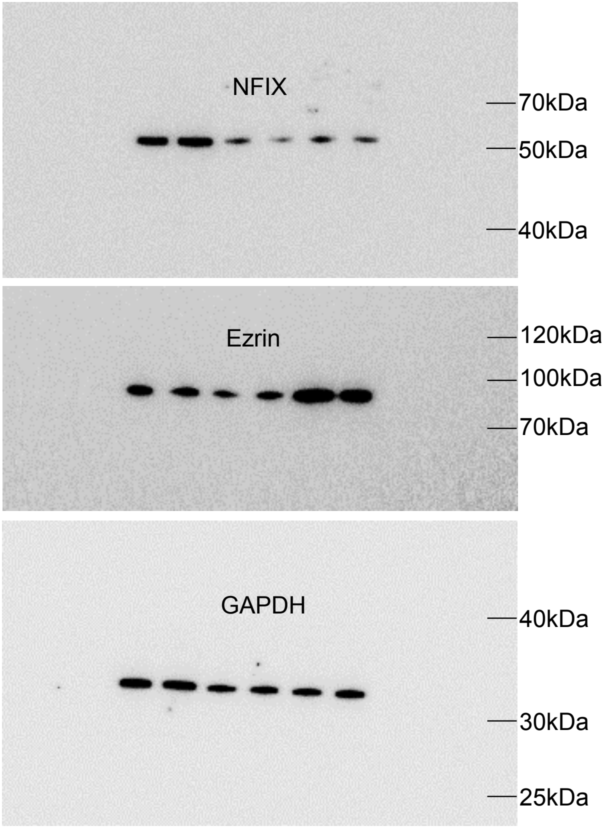

Figure S8. Full images of immunoblotting.

Gene name	Sequences (5’ to 3’)	
NFIA	Forward	AGCATGAGTCCAGGAGCAAT
	Reverse	TGACTGACTGCCACTTCCTG
NFIB	Forward	GCCACAATGATCCTGCCAAGAA
	Reverse	GGTGGAGAAGACAGAGACCTCTGA
NFIC	Forward	GGACAGGGATGGGCTCTG
	Reverse	CGTTCTTCTGAGGCCAGTGC
NFIX	Forward	CGGCTCTACAAGTCGCCTC
	Reverse	GCAGTGGTTTGATGTCCGC
Ezrin	Forward	AGCACACGGAGCACTGCAGG
	Reverse	GTAACTCGGACATTGATTGG
NIFX RE2	Forward	ACCCTCTGATACAGGTGCCA
	Reverse	TGGGGGCCAGTAGAATTTGC
Distal	Forward	TGCATAGAGGAAACCCAGCA
	Reverse	TGGAGGTTGCAGTGAGTCAA

Table S1. Primer sequences for real-time quantitative PCR analysis.

**siRNA**	**Sequences (5’ to 3’)**	
**siEzrin**	sense	AUCAGGUGGUAAAGACUAU
	antisense	AUAGUCUUUACCACCUGAU
**Control**	sense	UUCUCCGAACGUGUCACGU
	antisense	ACGUGACACGUUCGGAGAA

Table S2. Sequences of siRNAs.

GENETIC TESTING BIOTECHNOLOGY

Cell Line Authentication Service
STR Profile Report

Sample Submitted By: Dr. Zhuohao Liu
Shenzhen Hospital, Southern Medical University
Email Address: lchouhoo@gmail.com
Sales Order: 191220B
Cell Line Designation: U-87MG
Date Sample Received: Dec 20th, 2019
Report Date: Dec 20th, 2019

Methodology: Nineteen short tandem repeat (STR) loci plus the gender determining locus, Amelogenin, were amplified using the commercially available EX20 Kit from AGCU. The cell line sample was processed using the ABI Prism® 3130 XL Genetic Analyzer. Data were analyzed using GeneMapper® ID v3.2 software (Applied Biosystems). Appropriate positive and negative controls were run and confirmed for each sample submitted.

Data Interpretation: Cell lines were authenticated using Short Tandem Repeat (STR) analysis as described in 2012 in ANSI Standard (ASN-0002) by the ATCC Standards Development Organization (SDO) and in Capes-Davis et al., Match criteria for human cell line authentication: Where do we draw the line? Int J Cancer. 2013;132(11):2510-9.

GTB™ performs STR Profiling following ISO 9001:2008 and ISO/IEC 17025:2005 quality standards.
There are no warranties with respect to the services or results supplied, express or implied, including, without limitation, any implied warranty of merchantability or fitness for a particular purpose. Genetic Testing Biotechnology (GTB) is not liable for any damages or injuries resulting from receipt and/or improper, inappropriate, negligent or other wrongful use of the test results supplied, and/or from misidentification, misrepresentation, or lack of accuracy of those results. Your exclusive remedy against GTB and those supplying materials used in the services for any losses or damage of any kind whatsoever, whether in contract, tort, or otherwise, shall be, at GTB's option, refund of the fee paid for such service or repeat of the service.

NOTE: According to the recommendations of *IJC* on cell line authentication, the report is valid for 3 years since the issue date.

Technical Questions?
GTB Technical Support
+86-512-67486171
service@jsdna.org
Section 505, Yixin BLD
SIP, Suzhou, 215123
Jiangsu, P.R. China

Ordering Questions?
order@jsdna.org
GTB Corporation
+86-512-62806339
Section 303, Yixin BLD
SIP, Suzhou, 215123
Jiangsu, P.R. China

GENETIC TESTING BIOTECHNOLOGY

Cell Line Authentication Service
STR Profile Report

Sales Order: 191220B

Test Results for Submitted Sample			ExPASy Reference Database Profile	
**Loci**	**Query Profile:** U-87MG		**Database Profile:** U-87MG ATCC	
Amelogenin	X		X	
D3S1358	16	17		
D13S317	8	11	8	11
D7S820	8	9	8	9
D16S539	12		12	
Penta E	7	14		
TPOX	8		8	
TH01	9.3		9.3	
D2S1338	20	23		
CSF1PO	10	11	10	11
Penta D	9	14		
D19S433	15	15.2		
vWA	15	17	15	17
D21S11	28	32.2		
D18S51	13			
D6S1043	11	18		
D8S1179	10	11		
D5S818	11	12	11	12
D12S391	18	21		
FGA	18	24		

The allele match algorithm compares the 8 core loci plus amelogenin only, even though alleles from all loci will be reported when available.

Note: Loci highlighted in grey (8 core STR loci plus Amelogenin) can be made public to verify cell identity. In order to protect the identity of the donor, ***please do not publish*** *the allele calls from all the STR loci tested.*

Explanation of Test Results

Cell lines with ≥80% match are considered to be related; i.e., derived from a common ancestry. Cell lines with between a 55% to 80% match require further profiling for authentication of relatedness.

- ☐ The submitted sample profile is human, but not a match for any profile in the ExPASy STR database.
- ☑ The submitted profile is an exact match for the following human cell line(s) in the ExPASy STR database (8 core loci plus Amelogenin): U-87MG ATCC
- ☐ The submitted profile is similar to the following ExPASy human cell line(s):

e-Signature, Technician:

GENETIC TESTING BIOTECHNOLOGY – GUARANTEE – CELL LINE AUTHENTICATION ★★★★★

e-Signature, Reviewer:

More information

Addendum: Electropherogram for the customer's sample set 1 of 1

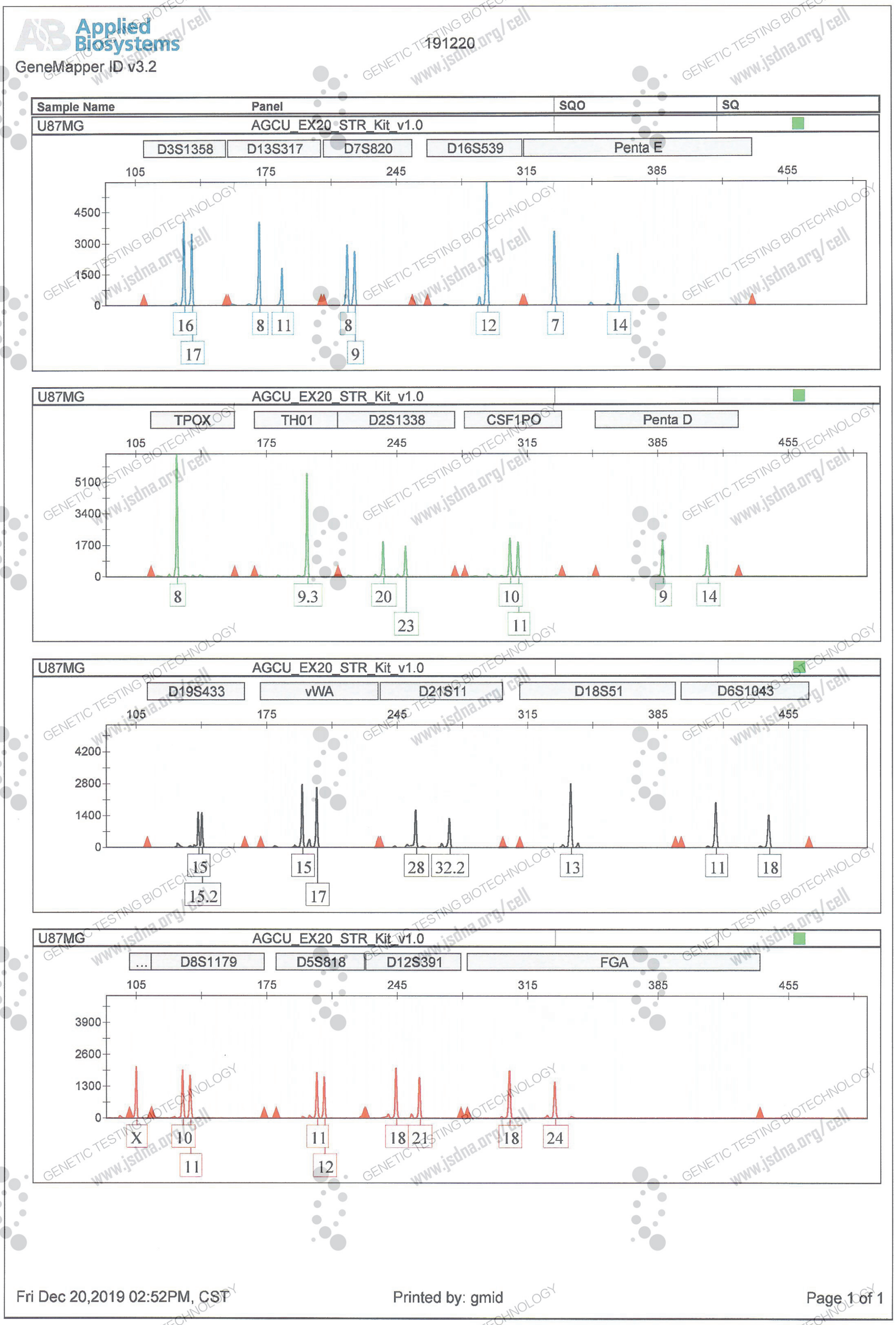
Applied Biosystems
GeneMapper ID v3.2
191220
Sample Name
Panel
SQO
SQ
U87MG
AGCU_EX20_STR_Kit_v1.0
D3S1358
D13S317
D7S820
D16S539
Penta E
16
17
8
11
8
9
12
7
14
TPOX
TH01
D2S1338
CSF1PO
Penta D
8
9.3
20
23
10
11
9
14
D19S433
vWA
D21S11
D18S51
D6S1043
15
15.2
15
17
28
32.2
13
11
18
D8S1179
D5S818
D12S391
FGA
X
10
11
11
12
18
21
18
24
Fri Dec 20,2019 02:52PM, CST
Printed by: gmid
Page 1 of 1

Cell Line Authentication Service
STR Profile Report

GENETIC TESTING BIOTECHNOLOGY

Sample Submitted By: Dr. Zhuohao Liu
Shenzhen Hospital, Southern Medical University
Email Address: lchouhoo@gmail.com
Sales Order: 191220C
Cell Line Designation: U-251MG
Date Sample Received: Dec 20th, 2019
Report Date: Dec 20th, 2019

Methodology: Nineteen short tandem repeat (STR) loci plus the gender determining locus, Amelogenin, were amplified using the commercially available EX20 Kit from AGCU. The cell line sample was processed using the ABI Prism® 3130 XL Genetic Analyzer. Data were analyzed using GeneMapper® ID v3.2 software (Applied Biosystems). Appropriate positive and negative controls were run and confirmed for each sample submitted.

Data Interpretation: Cell lines were authenticated using Short Tandem Repeat (STR) analysis as described in 2012 in ANSI Standard (ASN-0002) by the ATCC Standards Development Organization (SDO) and in Capes-Davis et al., Match criteria for human cell line authentication: Where do we draw the line? Int J Cancer. 2013;132(11):2510-9.

GTB™ performs STR Profiling following ISO 9001:2008 and ISO/IEC 17025:2005 quality standards.
There are no warranties with respect to the services or results supplied, express or implied, including, without limitation, any implied warranty of merchantability or fitness for a particular purpose. Genetic Testing Biotechnology (GTB) is not liable for any damages or injuries resulting from receipt and/or improper, inappropriate, negligent or other wrongful use of the test results supplied, and/or from misidentification, misrepresentation, or lack of accuracy of those results. Your exclusive remedy against GTB and those supplying materials used in the services for any losses or damage of any kind whatsoever, whether in contract, tort, or otherwise, shall be, at GTB's option, refund of the fee paid for such service or repeat of the service.

NOTE: According to the recommendations of *IJC* on cell line authentication, the report is valid for 3 years since the issue date.

Technical Questions?
GTB Technical Support
+86-512-67486171
service@jsdna.org
Section 505, Yixin BLD
SIP, Suzhou, 215123
Jiangsu, P.R. China

Ordering Questions?
order@jsdna.org
GTB Corporation
+86-512-62806339
Section 303, Yixin BLD
SIP, Suzhou, 215123
Jiangsu, P.R. China

GENETIC TESTING BIOTECHNOLOGY

Cell Line Authentication Service
STR Profile Report

Sales Order: 191220C

Test Results for Submitted Sample			ExPASy Reference Database Profile	
**Loci**	**Query Profile:** U-251MG		**Database Profile:** U-251MG	
Amelogenin	X		X	
D3S1358	16	17		
D13S317	10	11	10	11
D7S820	10	12	10	12
D16S539	12		12	
Penta E	7	10		
TPOX	8		8	
TH01	9.3		9.3	
D2S1338	22	24		
CSF1PO	11	12	11	12
Penta D	12			
D19S433	13	15		
vWA	16	18	16	18
D21S11	29			
D18S51	13			
D6S1043	12			
D8S1179	13	15		
D5S818	11	12	11	12
D12S391	17	22		
FGA	21	25		

The allele match algorithm compares the 8 core loci plus amelogenin only, even though alleles from all loci will be reported when available.

Note: Loci highlighted in grey (8 core STR loci plus Amelogenin) can be made public to verify cell identity. In order to protect the identity of the donor, ***please do not publish*** *the allele calls from all the STR loci tested.*

Explanation of Test Results

Cell lines with ≥80% match are considered to be related; i.e., derived from a common ancestry. Cell lines with between a 55% to 80% match require further profiling for authentication of relatedness.

- ☐ The submitted sample profile is human, but not a match for any profile in the ExPASy STR database.
- ☑ The submitted profile is an exact match for the following human cell line(s) in the ExPASy STR database (8 core loci plus Amelogenin): U-251MG
- ☐ The submitted profile is similar to the following ExPASy human cell line(s):

e-Signature, Technician: ______________________

e-Signature, Reviewer: ______________________

More information

Addendum: Electropherogram for the customer's sample set 1 of 1

Cell Line Authentication Service
STR Profile Report

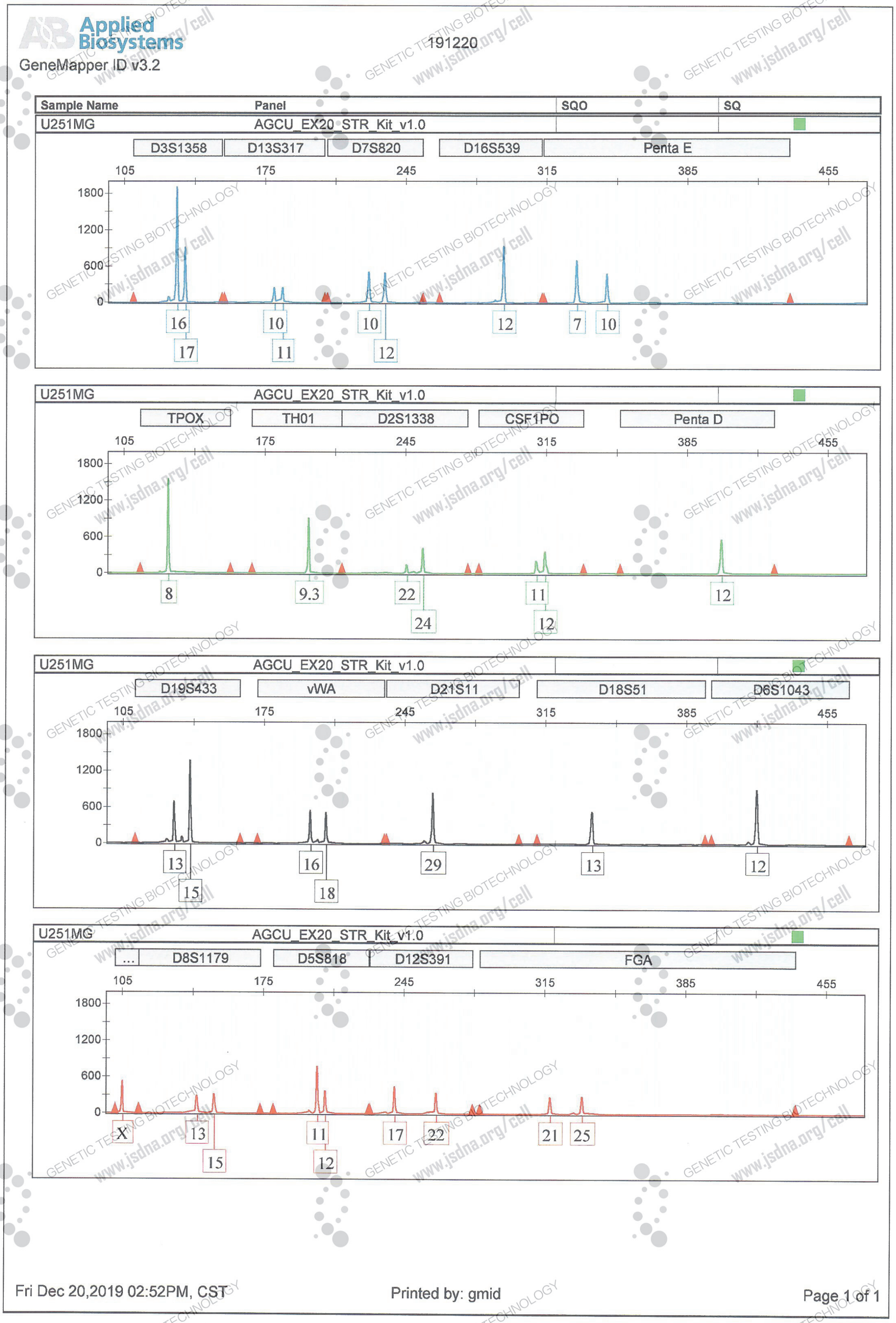

GENETIC TESTING BIOTECHNOLOGY www.jsdna.org/cell

Transcription Factor/Cis-element	Description	Normal 1	Normal 2	Normal 3	Normal 4	Normal 5	GBM 1	GBM 2	GBM 3	GBM 4	GBM 5	FC	Log2(FC)	p	-Log10(p value)
SRE	SREBF: sterol regulatory element binding transcription factor	0.03432716	0.026776	0.011079	0.012369	0.028163	0.036478	0.035703	0.048179	0.04125	0.038815	1.778173	0.830395	0.008993	2.046079524
NF-1	Nuclear Factor one family	0.03598074	0.088051	0.130074	0.69784	0.359343	0.948583	0.929698	0.375735	0.985284	0.911689	3.165582	1.662471	0.009414	2.026226309
E2F-1	E2F1: E2F transcription factor 1	0.75587116	0.835952	0.72903	0.825775	0.616765	0.904046	0.884607	0.827557	0.886921	0.924143	1.176405	0.234384	0.014504	1.838516813
NF-A3	Nuclear factor A3	0.75038984	0.683264	0.448053	0.809892	0.652805	0.816039	0.978576	0.696777	0.927296	0.840595	1.273555	0.348862	0.047896	1.319699055
HOXD8,9,10	homeo box D8, 9, 10	0.62438069	0.661657	0.667433	0.79864	0.38368	0.837272	0.962753	0.667536	0.812633	0.807263	1.303486	0.382375	0.04958	1.30469306
c-Myb	MYB: v-myb myeloblastosis viral oncogene homolog (avian)	0.57657989	0.556731	0.447375	0.714104	0.697528	0.75574	0.73348	0.580754	0.796552	0.868008	1.24804	0.319664	0.061417	1.211713737
NF-E2	NFE2: nuclear factor (erythroid-derived 2),	0.77825578	0.812351	0.733066	0.847025	0.315214	0.883395	0.968955	0.816467	0.915992	0.951992	1.301467	0.380139	0.071095	1.148163726
HFH-2	forkhead box D3	0.33432998	0.633074	0.41013	0.879635	0.085436	1.021085	0.996109	0.288256	0.988906	0.926176	1.80164	0.849311	0.089359	1.048862011
PTF1	pancreas specific transcription factor	0.13326654	0.411235	0.035539	0.696805	0.396834	0.085093	0.125247	0.133477	0.176897	0.097636	0.369455	-1.43653	0.11011	0.958174617
CBF	mouse CCAAT-binding factor, CP1 (human, rat); NF-Y	0.07389066	0.202068	0.021155	0.261673	0.376016	0.02201	0.204834	0.017302	0.013271	0.017864	0.29448	-1.76376	0.113064	0.94667487
AhR/Arnt	aryl hydrocarbon receptor/aryl hydrocarbon receptor nuclear translocator binding element	0.62153285	0.548573	0.759016	0.880098	0.348823	0.940264	0.965837	0.618903	0.710367	0.91463	1.314105	0.394081	0.121013	0.917169438
NF-E1 (YY1)	YY1 transcription factor	0.84865554	0.909712	0.67472	0.755432	0.358777	0.837758	0.913807	0.788741	0.952215	0.899162	1.238037	0.308054	0.131885	0.879805119
TFEB	TFEB is closely related to TFE3 leucine zipper: essential for dimerization; free in solution: tetramerization:	0.63849739	0.544339	0.281411	0.869337	0.14443	0.685503	0.917864	0.745774	0.639484	0.650153	1.468425	0.554269	0.132267	0.878549282
MRE	Metal response factor	0.78633996	0.874778	0.710232	0.913689	0.506107	0.908674	0.935598	0.803831	0.805264	0.914194	1.152043	0.204194	0.176062	0.754335141
Tat	transactivating regulatory protein	0.48753139	0.689803	0.234902	0.590256	0.793317	0.346231	0.541519	0.374796	0.515859	0.100577	0.672071	-0.57332	0.17704	0.751929539
NFkBp65		0.05567063	0.091974	0.044288	0.072413	0.318353	0.044634	0.064635	0.031998	0.021952	0.040303	0.349276	-1.51756	0.179735	0.745367036
MEF-1	MEF-1Myocyte enhancing factor1	0.82614844	0.886142	0.814924	0.934449	0.770203	0.913982	0.999428	0.805558	0.935009	0.889504	1.073636	0.102504	0.182702	0.738256312
Sp1	SP1: Sp1 transcription factor	0.60493578	0.641232	0.760316	0.861818	0.64769	0.94567	0.96349	0.585299	0.735879	0.90174	1.175224	0.232936	0.192623	0.715291656
TEF1(1)	activator; function depends on a highly limiting cofactor. interference with TEF-1 / TFIID interaction by a B-cell specific factor	0.43636218	0.690799	0.284336	0.450633	0.386799	0.166691	0.434275	0.389977	0.465241	0.08195	0.68394	-0.54806	0.200884	0.697054319
ISRE (TRANSFAC)	interferon-a stimulated response element	0.95690398	0.981447	0.957025	0.98899	0.786293	0.985222	1.040529	0.95355	1.006455	0.963974	1.05975	0.083724	0.206689	0.684682171
CREB	CREB1: cAMP responsive element binding protein 1	0.2973081	0.230899	0.268572	0.592299	0.552193	0.63692	0.679841	0.241228	0.451626	0.752473	1.422824	0.508757	0.20725	0.6835046
ADD1	sterol regulatory element binding transcription factor	0.7557793	0.629899	0.802193	0.910638	0.132115	0.863587	1.01485	0.788378	0.665745	0.83809	1.290973	0.368459	0.238065	0.623305292
Stat5b	MGF; signal transducer and activator of transcription 5; mammary gland factor; STF-	0.1290101	0.134193	0.034401	0.211763	0.801396	0.050752	0.20061	0.073662	0.048338	0.040412	0.315674	-1.66349	0.238801	0.621964549
ERE	ESR: estrogen receptor	0.72028851	0.853232	0.564339	0.699257	0.200391	0.674757	0.749303	0.609722	0.945002	0.867863	1.266383	0.340714	0.239631	0.620457885
ISGF	Interferon-Stimulated Response factor	0.40886371	0.807712	0.771395	0.886555	0.732433	0.68793	1.0413	0.205927	0.172119	0.323334	0.673867	-0.56946	0.240158	0.619502694
lactoferrin BP		0.6621375	0.259824	0.586767	0.845554	0.294602	0.787233	0.97281	0.560512	0.424178	0.85156	1.357664	0.441126	0.240811	0.618323943
p53	TP53: tumor protein p53	0.71425599	0.814936	0.473217	0.704215	0.65439	0.724376	0.877534	0.538059	0.890418	0.887979	1.165829	0.221356	0.245201	0.610477263
Mfh-1	Mesenchyme fork head 1; Fkh-14; Fork head homologue 14;	0.36939207	0.320881	0.072919	0.074647	0.805889	0.041139	0.092326	0.326072	0.231043	0.049417	0.450194	-1.15138	0.248726	0.604278264
HOXD8	homeo box D8	0.03729748	0.290928	0.034943	0.307333	0.831947	0.032853	0.373528	0.024362	0.015457	0.065829	0.340796	-1.55302	0.252375	0.597954463
AP-1	Fos, FosB, Fra1, Fra2, Jun, JunB	0.65521695	0.697493	0.516692	0.835147	0.558107	0.746483	0.885881	0.645144	0.628804	0.813763	1.140198	0.189284	0.253939	0.59527005
ATF/CRE		0.03898169	0.173019	0.021534	0.484006	0.938048	0.040297	0.385094	0.018938	0.033443	0.052431	0.320251	-1.64273	0.262025	0.581657125
Fkhr	forkhead box O1mouse	0.92141319	0.815994	0.843502	0.827682	0.137129	0.942983	0.918601	0.789044	0.874149	0.90439	1.249159	0.320957	0.262615	0.580680225
TTF1	traT/EBP, thyroid-specific enhancer-binding protein, thyroid nuclear nscriptional intermediary factor 1	0.0655615	0.267764	0.026654	0.481827	0.830761	0.074962	0.424251	0.049482	0.021265	0.110562	0.406873	-1.29735	0.264825	0.577040528
pax2	Pax-2DNA-binding transcription factor	0.68326662	0.575816	0.600203	0.875712	0.731874	0.812253	0.984208	0.631932	0.687884	0.840305	1.141254	0.19062	0.264876	0.576956648
PBGD binding protein	porphobilinogen deaminase	0.20017541	0.130831	0.334638	0.754315	0.109866	0.752665	0.987225	0.060724	0.233011	0.712823	1.795269	0.8442	0.279691	0.553322016
MSP1	the sequences are the same as SAA except SP1 binding site is removed	0.86813107	0.969678	0.643678	0.86694	0.017005	0.786132	1.005597	0.860616	0.989218	0.762167	1.308519	0.387934	0.281133	0.551088383
PRDII-BF1	alphaA-crystallin binding protein I	0.57942773	0.718665	0.179237	0.844437	0.959474	0.310562	0.88977	0.501395	0.357947	0.174647	0.680938	-0.5544	0.283834	0.546935984
Pax8	paired box gene 8	0.99080244	0.986367	0.950984	0.9718	0.416199	0.969168	1.016425	1.011516	0.993559	0.97011	1.149352	0.200821	0.284577	0.545800114
v-Maf	v-maf musculoaponeurotic fibrosarcoma oncogene homolog (avian)	0.05273092	0.076904	0.027169	0.063777	0.680161	0.023693	0.048007	0.045482	0.03616	0.033913	0.207889	-2.26611	0.287883	0.540784295
Tf-LF		0.44227221	0.470019	0.255326	0.713805	0.0254	0.563511	0.855676	0.450549	0.418432	0.42431	1.422514	0.508442	0.288082	0.540483508
EBP40, 45		0.93439688	0.913168	0.851655	0.981199	0.515457	0.895145	1.013274	0.902129	0.985409	0.89564	1.118145	0.161107	0.289068	0.538999621
WT1 (2)	Wilms tumor 1	0.89376161	0.945175	0.885189	0.88176	0.218238	0.960429	1.011531	0.809589	0.961458	0.873273	1.207147	0.271601	0.296287	0.528287132
E4BP4	nuclear factor, interleukin 3 regulated	0.48394862	0.692543	0.452522	0.842421	0.093834	0.843454	0.992656	0.253258	0.63583	0.846441	1.392307	0.477477	0.297078	0.52712904
E47	E2A immunoglobulin enhancer binding factors E12/E47(basic helix-loop-helix	0.92677203	0.986024	0.989746	1.002884	0.59245	0.984187	1.030472	0.960277	1.010421	0.953553	1.098053	0.134948	0.298038	0.52572847
ODC	ODC (ornithine decarboxylase	0.44077173	0.62227	0.504719	0.851929	0.568272	0.609667	0.996914	0.518757	0.766918	0.692236	1.199644	0.262607	0.299831	0.523123679
MEF-2a	MADS box transcription enhancer factor 2	0.08785425	0.075908	0.028306	0.321445	0.951299	0.03337	0.34215	0.041028	0.029915	0.004793	0.308064	-1.6987	0.301424	0.52082186
RB	retinoblastoma tumor suppressor protein	0.0683481	0.585064	0.035159	0.410286	0.320206	0.051917	0.488718	0.062057	0.035036	0.035982	0.474757	-1.07474	0.307082	0.512745535
Pax4	paired box gene 4	0.98578044	0.960991	0.970731	0.991905	0.520684	0.971272	1.020917	0.97367	1.002521	0.961614	1.112842	0.154249	0.309616	0.509176798
Stat1/Stat3	Stat1 (STAT91; ISGF3alpha p91; signal transducer and activator of transcription 1) Stat3 (signal transducer and activator of transcription 3; APRF; acute-phase)	0.27706702	0.635845	0.077009	0.671196	0.889413	0.231587	0.842367	0.159748	0.134336	0.086598	0.570326	-0.81014	0.310211	0.508342849
EGR	early growth response	0.9834838	0.982101	1.005782	0.981634	0.401001	0.997133	1.020415	0.974336	0.998586	1.000973	1.146403	0.197115	0.31055	0.507867869
Antioxidant RE	Antioxidant responsive element	0.9416849	0.976621	0.955156	0.967059	0.127958	0.979849	1.00848	0.956247	0.985783	0.940554	1.2274	0.295606	0.311187	0.506979125
GRE	GR:glucocorticord receptor	0.79791504	0.950499	0.537631	0.817684	0.030683	0.688092	0.975827	0.702837	0.976946	0.741798	1.303435	0.382318	0.31144	0.506625068
WT1 (1)	Wilms tumor 1	0.9755221	0.998229	0.950741	0.946381	0.077862	0.955736	1.011698	0.965791	0.999179	0.973814	1.242478	0.313221	0.314705	0.502096328
msx1/2/3	Ghox-8 (chick); Hox-8; Chox-8 (chick); Quox-7 (quail); Hox-8.1;	0.04614721	0.07432	0.025245	0.065847	0.185472	0.04512	0.090784	0.029029	0.023201	0.047674	0.593925	-0.75165	0.31766	0.498038134
GATA-1/2	GATA binding protein 1/2 (globin transcription factor)	0.9453289	0.999786	1.014531	1.011929	0.348407	0.992408	1.044217	0.969185	1.01701	0.990153	1.160415	0.214641	0.317976	0.497605021
LyF	LyF	0.03500084	0.887574	0.018365	0.718436	0.821424	0.022236	0.957154	0.019302	0.022108	0.030064	0.423599	-1.23923	0.3187	0.496617468
HFH-3	forkhead box I1	0.64584665	0.733735	0.864846	0.985966	0.705807	0.948259	1.051291	0.646022	0.88614	0.885074	1.122094	0.166193	0.319141	0.496017363
XRE	xenobiotic response element	0.86007751	0.980171	0.680165	0.927011	0.799495	0.610703	1.00439	0.819861	0.782032	0.480407	0.870606	-0.19991	0.321686	0.492567868
GATA-2	GATA binding protein 2 (globin transcription factor 2)	0.9671317	0.996455	1.010062	1.003429	0.45078	1.00516	1.031545	0.960489	1.018227	0.991351	1.130744	0.177272	0.321744	0.492489398
Pax6	paired box gene 6	0.98547422	0.987519	0.9473	0.987982	0.023507	0.961497	1.02313	0.989214	0.988906	0.976428	1.256218	0.329087	0.322632	0.49129283
RAR(DR-5)	RAR: retinoic acid receptor	0.79607773	0.90968	0.58739	0.891077	0.068783	0.748716	0.955813	0.688202	0.931699	0.795281	1.266431	0.340768	0.323339	0.490341814
PAX1	Pax-1DNA-binding transcription factor	0.71345982	0.74731	0.538064	0.808475	0.751845	0.721269	0.933687	0.814013	0.713459	0.70498	1.092228	0.127274	0.331445	0.479589158
Ets	ETS: v-ets erythroblastosis virus E26 oncogene homolog (avian)	0.69768954	0.955076	0.694602	0.813052	0.543403	0.820765	0.785141	0.72026	0.881675	0.884929	1.105012	0.144062	0.331519	0.479491425
GATA-1	GATA binding protein 1 (globin transcription factor 1)	0.94263417	0.948101	0.988988	1.004819	0.607522	0.993961	1.035601	0.951489	0.989281	0.916155	1.087804	0.121419	0.332135	0.478685696
HOXA4	Chox-1.4; Chox1.4; Hox-1.4 (mouse);	0.08448584	0.064761	0.032424	0.13856	0.591761	0.086841	0.187669	0.043149	0.023326	0.012018	0.387069	-1.36934	0.333317	0.477142717
HiNF-B/H1TF1	Human H1 Histone Gene Promoter CCAAT Box Binding Protein HiNF-B Is a Mosaic	0.11097379	0.768762	0.038681	0.694026	0.73027	0.112314	0.884004	0.069905	0.030133	0.070222	0.497961	-1.0059	0.335663	0.474096855
SIF2	SI promoter 2	0.03248984	0.695843	0.011702	0.430664	0.538334	0.003722	0.689294	0.017605	0.004684	0.005301	0.421646	-1.2459	0.336644	0.472829422
TFIID	TBP: TATA box binding protein	0.93228397	0.972729	0.935572	0.981771	0.688384	0.925732	1.054006	0.906219	1.007735	0.929298	1.069224	0.096564	0.337852	0.471273908
CCAC	CCAC binding protein	0.96263028	0.972885	0.937495	0.961393	0.129287	0.955639	1.026617	0.942884	0.999242	0.889431	1.214477	0.280336	0.340185	0.468284457
c-fos enhancer-binding protein	c-fos enhancer-binding protein	0.38801019	0.376457	0.064739	0.072685	0.451659	0.038679	0.06326	0.431702	0.212308	0.034312	0.576455	-0.79472	0.340228	0.468230181
XBP1(2)	X-box binding protein 1; TREB-5; hXBP-1	0.79975236	0.912763	0.520295	0.840977	0.472585	0.813094	0.87978	0.6669	0.865843	0.811148	1.13828	0.186856	0.340269	0.468178192
RORE	RAR-related orphan receptor	0.09523413	0.446325	0.021914	0.403475	0.463957	0.061077	0.532233	0.075359	0.028791	0.055698	0.526351	-0.9259	0.342214	0.465702132
IL-6 RE-BP	interleukin-6 response element binding	0.38993937	0.324243	0.078987	0.071732	0.341629	0.06399	0.06782	0.347404	0.230637	0.036273	0.618404	-0.69338	0.342683	0.465107185
AP-2	AP2 (activating enhancer binding protein 2)	0.83956083	0.920765	0.64449	0.814632	0.13541	0.76545	0.941096	0.779075	0.888919	0.719903	1.220452	0.287416	0.344227	0.463155007
EIL1/2/3	ETHYLENE-INSENSITIVE3-LIKE1/2/3	0.37713941	0.461488	0.069967	0.257941	0.594154	0.062307	0.479801	0.350282	0.225173	0.028358	0.650837	-0.61963	0.348815	0.457405249
HMG	high mobility group proteins	0.93265143	0.994306	0.870291	0.96676	0.176724	0.899838	1.025142	0.90834	0.979788	0.899198	1.195794	0.257969	0.35258	0.452742658
EGR1	Early growth factor1	0.75516686	0.780811	0.754438	0.745325	0.040932	0.861289	0.944415	0.542877	0.80592	0.707304	1.255188	0.327903	0.353641	0.451437302
c-Rel	NFkB p75kDa protein	0.86436458	0.880008	0.935572	0.974987	0.660816	0.971887	1.020582	0.80668	0.947156	0.891428	1.074607	0.103809	0.354566	0.450302344
RXR(DR-1)	RXR: retinoid X receptor	0.21132178	0.122611	0.393336	0.796052	0.455391	0.730655	0.826174	0.147355	0.375621	0.761187	1.435779	0.521833	0.354854	0.449950783
CP1B	CBF-B (rat); NF-YA (mouse); HAP2 (yeast); CP1-B	0.03405156	0.040632	0.030338	0.040021	0.623829	0.038031	0.077207	0.031422	0.010867	0.03217	0.246722	-2.01904	0.355132	0.449609798
NF-Y	nuclear Y box factor	0.7837371	0.954391	0.560249	0.911701	0.030635	0.775678	1.009921	0.799135	0.932823	0.61595	1.275493	0.351055	0.355559	0.449088107
myc-PRF	A transcriptional repressor of c-myc	0.06648016	0.057725	0.023458	0.047349	0.363966	0.050363	0.100875	0.028513	0.017705	0.04397	0.431909	-1.2112	0.357521	0.44669902
ATF2		0.36979015	0.17333	0.280084	0.565682	0.833671	0.369988	0.612859	0.237077	0.128153	0.163428	0.680075	-0.55623	0.359022	0.444879004
alpha-PAL	Eukaryotic Initiation Factor 2alpha	0.02465064	0.080049	0.054662	0.088922	0.076068	0.044505	0.030205	0.032847	0.015114	0.106422	0.706308	-0.50163	0.359568	0.444218785
AP3	activator protein 3 binding element	0.94557388	0.978085	0.946759	0.960194	0.647496	0.948097	1.003485	0.964519	0.982317	0.893461	1.070068	0.097702	0.362892	0.440222122
ADR1	alcohol dehydrogenase regulatory gene1binding element	0.94514517	0.983378	0.906019	0.904372	0.667534	0.903949	0.98736	0.917976	0.998118	0.885437	1.064993	0.090844	0.36679	0.435582986
CBFB	CCAAT-binding factor	0.03356161	0.050813	0.022022	0.190431	0.874689	0.041236	0.292265	0.010636	0.000343	0.002941	0.296557	-1.75362	0.366942	0.435402022
NFkB(2)		0.90536731	0.896541	0.594839	0.916713	0.054256	0.807915	0.954103	0.795105	0.894509	0.722953	1.239589	0.309862	0.372982	0.42831155
GAS	gamma-interferon activation site	0.0324286	0.029298	0.025056	0.068762	0.643855	0.06386	0.08723	0.023817	0.027448	0.021786	0.280386	-1.83451	0.373078	0.428199922
EKLF(2)	erythroid Kruppel-like factor gene	0.19460222	0.380038	0.330954	0.80289	0.082617	0.634589	0.928122	0.189746	0.42958	0.424128	1.455063	0.541082	0.377101	0.423542272
PYR	pyrimidine-rich domain binding factor	0.54966324	0.636126	0.440062	0.811935	0.786352	0.772182	0.957825	0.505849	0.88614	0.613517	1.158608	0.212393	0.377267	0.423350907
HNF-3 (a, b, g)	hepatocyte nuclear factor 3 (a, b, g)	0.00624687	0.036989	0.013056	0.052035	0.636495	0.043437	0.081498	0.008272	0.017393	0.022076	0.231835	-2.10883	0.378901	0.421474512
PPAR	PPAR: peroxisome proliferative activated	0.66388295	0.764528	0.828414	0.953383	0.559808	0.936024	0.99336	0.584723	0.848637	0.856063	1.119042	0.162265	0.383317	0.416441708
TR(DR-4)	thyroid hormone receptor	0.39545131	0.430882	0.542723	0.906824	0.673428	0.828792	0.94589	0.472487	0.509145	0.800038	1.205826	0.270022	0.383856	0.415832054
CBF	CAAT box General	0.76478214	0.751762	0.711721	0.851711	0.805262	0.73373	0.901772	0.724987	0.840893	0.88856	1.052688	0.074077	0.385014	0.414522987
Angiotensinogen ANG-IRE	Angiotensinogen(ANG) insulin-response element (IRE)	0.13951341	0.265397	0.331143	0.591809	0.343865	0.638441	0.839852	0.110084	0.199474	0.60444	1.43103	0.517054	0.38629	0.413085981
HNF-3 b	hepatocyte nuclear factor 3 beta (forkhead domain)	0.82431112	0.863849	0.776	0.967086	0.26275	0.786585	1.009452	0.868949	0.956712	0.692054	1.167774	0.223761	0.387788	0.411405627
E12	E2A immunoglobulin enhancer binding factors E12	0.96633553	1.007445	0.990803	1.019149	0.296631	0.952564	1.045927	0.949035	1.01854	0.960488	1.150966	0.202845	0.387833	0.411355293

Transcription Factor/Cis-element	Description	Normal 1	Normal 2	Normal 3	Normal 4	Normal 5	GBM 1	GBM 2	GBM 3	GBM 4	GBM 5	FC	Log2(FC)	p	-Log10(p value)
NFIL-2	5' upstream activating sequences (UAS) of the human IL-2 gene	0.26996274	0.918056	0.085975	0.828063	0.425892	0.067971	1.005128	0.112539	0.159442	0.083983	0.565305	-0.8229	0.388851	0.410216445
YB1	DbpB; EFIA (rat, chick);	0.79662892	0.960306	0.279244	0.733556	0.261286	0.105549	0.783531	0.6126	0.524383	0.118332	0.707483	-0.49923	0.393524	0.405028753
CREB(2)	ATF-47 (human); CREB-341; CREBalpha; CREB-B	0.03784868	0.077776	0.021616	0.041328	0.363972	0.030555	0.154648	0.012969	0.008244	0.020261	0.417804	-1.2591	0.394274	0.40420139
SIF3	SI promoter 3	0.03601136	0.454233	0.010456	0.751727	0.897979	0.016831	0.949276	0.01209	0.006807	0.018844	0.466818	-1.09907	0.403973	0.393647892
Stat4	STAT4: signal transducer and activator of transcription 4	0.79105573	0.927583	0.504638	0.789568	0.015573	0.643231	0.719265	0.678414	0.918928	0.810604	1.24502	0.316169	0.408826	0.388461346
PARP	poly(ADP-ribose) synthetase/polymerase	0.54216087	0.870232	0.799647	0.899577	0.228778	0.939099	1.023499	0.430247	0.859223	0.788237	1.209529	0.274445	0.414844	0.382114934
CCAAT	CCAAT binding protein	0.59287075	0.519275	0.516448	0.806378	0.202644	0.799014	0.969927	0.345707	0.512861	0.626153	1.233562	0.30283	0.422181	0.374501507
PO-B	Stimulatory factor, binds at -15 in proopiomelanocortin (POMC) gene; unusal location for a promoter binding site; candidate factor, cloned using PO-B binding site, is a phosphoprotein that resembles DNA ligases and repair enzymes	0.06850121	0.072545	0.02809	0.275376	0.864868	0.070042	0.448657	0.030725	0.018361	0.023093	0.451266	-1.14795	0.441503	0.355066639
CREB-BP1	cAMP response element binding protein	0.02633484	0.021483	0.016198	0.03588	0.285807	0.05347	0.0526	0.017908	0.018142	0.030137	0.446607	-1.16292	0.442687	0.353903501
TREF1, 2	transferrin receptor (TR) binding protien	0.06522466	0.085622	0.01999	0.706585	0.97049	0.094382	0.599181	0.07339	0.033194	0.17944	0.530105	-0.91565	0.456395	0.34065941
NF-1(2)	CTF; NF-I; TGGCA-binding protein	0.22338681	0.333833	0.20321	0.721651	0.038566	0.510818	0.875422	0.163415	0.044935	0.666129	1.486683	0.572097	0.463718	0.333745627
NF-Gma (1)		0.19585772	0.481912	0.216455	0.57519	0.881007	0.326616	0.859799	0.0573	0.04559	0.314765	0.682461	-0.55118	0.464493	0.333020407
NZF-3	neural zinc finger factor 3	0.4277268	0.689554	0.291406	0.831605	0.182599	0.550888	0.98166	0.303194	0.614065	0.59558	1.256924	0.329897	0.466416	0.331226168
PEBP2	polyoma enhancer binding protein	0.91783043	0.939134	0.826139	0.87247	0.988859	0.864979	1.01314	0.904674	0.883736	0.591877	0.93706	-0.09379	0.468736	0.329071586
CPE	Cap proximal element	0.05518068	0.164208	0.028767	0.340107	0.717871	0.032367	0.547386	0.026089	0.045247	0.035837	0.525924	-0.92707	0.469025	0.328803925
Surf-2(2)	Surfeit	0.89758935	0.937048	0.804279	0.918375	0.157909	0.863554	0.955076	0.73123	0.942066	0.806537	1.156993	0.210381	0.470165	0.327749489
RFX1,2,3,	Regulatory Factor X (RFX)	0.28995884	0.899748	0.075276	0.784147	0.989244	0.192908	0.96349	0.386492	0.381304	0.265275	0.720606	-0.47272	0.473359	0.324809528
ARE	androgen receptor	0.97931922	1.002308	0.977801	1.033288	0.681734	0.977228	1.059605	0.959216	1.009109	0.925087	1.054722	0.076863	0.473649	0.324543357
CEF2	cTnC (Slow/Cardiac Troponin C)	0.95359682	0.99524	0.975688	1.018277	0.745818	0.942206	1.032014	0.936975	1.020788	0.956276	1.04258	0.060157	0.474043	0.324181828
MyoD	myogenic factor D	0.26506323	0.225918	0.445886	0.859393	0.690967	0.665565	0.95635	0.493638	0.263675	0.738203	1.253376	0.325819	0.477006	0.321476228
SP1, ASP		0.22295811	0.707581	0.12639	0.734401	0.214553	0.139405	0.927385	0.043937	0.030539	0.060564	0.599153	-0.739	0.480374	0.318420469
SRF(2)	serum response factor; CArG-binding factor; CBF (3) (mouse); p67; p67SRF	0.16612385	0.167726	0.033263	0.545195	0.502554	0.142512	0.543631	0.053391	0.061453	0.112885	0.64591	-0.6306	0.484896	0.314351576
ZNF174	zinc-finger protein 174	0.04562664	0.569122	0.018934	0.780442	0.516176	0.038582	0.962853	0.02012	0.003091	0.02538	0.54397	-0.8784	0.486669	0.312766737
HIF-1	hypoxia-inducible factor 1	0.10420635	0.190859	0.685149	0.89451	0.581737	0.851514	1.003183	0.05736	0.460682	0.924687	1.342348	0.424759	0.489007	0.310684599
SIE	serum inducible element responsive factor	0.10840155	0.06526	0.282711	0.753825	0.538637	0.662004	0.840724	0.05127	0.295401	0.594673	1.397543	0.482893	0.489359	0.310372432
Pur-1	MYC-associated zinc finger protein (purine-binding transcription factor)	0.01895496	0.027648	0.02083	0.352775	0.465389	0.100241	0.310536	0.017484	0.012397	0.045641	0.549119	-0.86481	0.493225	0.306954814
TFE3-L	TFE3-L is an approximately 3-fold stronger activator than TFE3-S; cooperating with ITF-1, TFE3 can mediate lymphoid-specific activation through combined elements (such as muE5 and muE3 in the IgH enhancer)	0.547091	0.538423	0.104612	0.944556	0.400076	0.465018	0.975391	0.54521	0.540309	0.590533	1.229491	0.298061	0.495322	0.305112758
p300	p300 coactivator	0.025998	0.025811	0.018067	0.034082	0.321541	0.026056	0.14211	0.011151	0.011772	0.007806	0.467439	-1.09715	0.502429	0.298925061
MUSF1	the sequences are the same as SAA except USF binding site is removed	0.7805218	0.844359	0.823403	0.922979	0.879832	0.875239	1.037378	0.738229	0.926484	0.863724	1.044685	0.063068	0.503231	0.298233039
HOXD8	homeo box D8	0.01007461	0.054331	0	0.063613	0.331442	0.018676	0.139227	0.014999	0.003529	0.05214	0.497474	-1.00731	0.503617	0.297899828
EGR2 BP	Early growth factor2 binding protein	0.10215468	0.189801	0.244139	0.843892	0.158575	0.755513	0.939587	0.089995	0.043998	0.490756	1.507804	0.592449	0.504083	0.29749807
ICSBP	interferon consensus sequence binding	0.13856413	0.836295	0.071917	0.844164	0.577145	0.123545	0.971	0.08951	0.307236	0.167676	0.672167	-0.57311	0.507701	0.294391982
PREB	a Growth Hormone Gene Promoter	0.08917099	0.149232	0.080937	0.623738	0.759447	0.304413	0.582888	0.055724	0.0662	0.076794	0.637887	-0.64863	0.50781	0.29429849
Myc/Max	myc-associated factor X	0.00869662	0	0.003548	0.034327	0.803902	0.069233	0.125079	0.010636	0.032756	0.059511	0.349471	-1.51676	0.508114	0.294038465
ALF1B	murine leukemia virus	0.44772291	0.757522	0.107564	0.610797	0.631732	0.169345	0.782794	0.629448	0.276197	0.091753	0.762927	-0.39038	0.508915	0.293355011
Myb(2)		0.14664831	0.411609	0.632328	0.846262	0.654639	0.908189	1.032249	0.079632	0.58618	0.794954	1.26369	0.337642	0.509442	0.292905206
N-ras binding protein	N-ras promoter region	0.17194201	0.200792	0.371504	0.855933	0.070803	0.520366	1.01381	0.075117	0.168591	0.644852	1.449895	0.535948	0.512298	0.290477009
SSAP	Novel activator of sea urchin H1 gene; related to several RNA-binding protien and	0.09768388	0.084626	0.036974	0.227755	0.015788	0.086841	0.818799	0.047512	0.02573	0.028031	2.175573	1.121395	0.513323	0.289609579
KTP1	keratinocyte transcriptional protein-1	0.01267747	0.023507	0.014736	0.017626	0.64485	0.014824	0.030138	0.011454	0.101547	0.120549	0.390403	-1.35696	0.514864	0.288307544
HLF	hepatic leukemia factor	0.02103725	0.127655	0.01953	0.233966	0.346594	0	0.354319	0.007878	0.055645	0.01594	0.579317	-0.78757	0.515105	0.288104227
EKLF(1)	erythroid Kruppel-like factor gene	0.16771619	0.827359	0.069181	0.857486	0.74052	0.205984	0.98803	0.168869	0.314824	0.205474	0.707361	-0.49948	0.518138	0.285554483
AP3	activator protein 3	0.03187741	0.062426	0.013923	0.637414	0.538485	0.052273	0.559555	0.033028	0.012085	0.047456	0.548542	-0.86632	0.519239	0.284632312
PPUR(1)	purine-rich sequences binding sequence	0.2665637	0.291489	0.851465	0.936874	0.134525	0.922786	1.025443	0.336889	0.10033	0.933765	1.337898	0.419968	0.519866	0.284108854
H4TF-1	Histone 4 binding protein	0.58760378	0.634008	0.666323	0.779434	0.282609	0.885791	0.995137	0.337616	0.453625	0.787329	1.17272	0.229859	0.52015	0.283871134
PPARr	peroxisome proliferator activated receptor	0.00290908	0.031478	0.011079	0.049774	0.650917	0.027383	0.099467	0.104	0.01043	0.080498	0.431245	-1.21342	0.523184	0.281345768
TGT3	TTF-1 (Thyroid Transcription Factor 1)binding element	0.01353488	0.064014	0.070211	0.588022	0.819915	0.162094	0.588956	0.029029	0.009774	0.112595	0.580092	-0.78565	0.523468	0.281109643
Pbx1	PBX1: pre-B-cell leukemia transcription	0.34740554	0.439786	0.635037	0.880779	0.356566	0.815878	0.995707	0.178717	0.454343	0.803959	1.221475	0.288624	0.527649	0.277654516
USF-1	USF: upstream transcription factor	0.78027682	0.866869	0.501334	0.838661	0.720369	0.787233	0.851351	0.671142	0.887826	0.756285	1.06644	0.092803	0.53128	0.274676138
EBP-80		0.03490897	0.145682	0.027602	0.72274	0.563106	0.116036	0.707867	0.017666	0.004778	0.005374	0.570079	-0.81077	0.535136	0.271536101
GAS/ISRE	interferon activated factors	0.90300943	0.91432	0.798238	0.838443	0.37424	0.810149	0.974654	0.724927	0.886171	0.785369	1.092214	0.127256	0.535483	0.271254636
HNF-4 (2)	hepatic nuclear factor 4	0.01506598	0.037829	0.137848	0.705468	0.160331	0.844911	0.869521	0.009424	0.016706	0.085073	1.727934	0.789048	0.535986	0.270846852
CSBP	conserved sequence-binding protein 1	0.06482658	0.341305	0.04976	0.624719	0.873873	0.066191	0.857587	0.03118	0.084155	0.195779	0.631825	-0.6624	0.5367	0.270268551
SPERM1	A Pou domain gene transiently expressed immediately prior to meiosis I in the male germ cell	0.07722845	0.069992	0.034374	0.299051	0.149727	0.201129	0.826409	0.052451	0.03285	0.032279	1.816575	0.861221	0.53734	0.269750543
WAP BP	whey acidic protein	0.3497328	0.404354	0.098544	0.454829	0.518699	0.123934	0.732876	0.268075	0.199599	0.056933	0.75646	-0.40266	0.542049	0.265961244
MEF-2	MEF-2Myocyte enhancing factor2	0.07297201	0.090199	0.444315	0.788261	0.214773	0.607595	0.949712	0.029695	0.093117	0.631018	1.435026	0.521077	0.542412	0.265670403
myc-CF1	common factor 1; CF1;	0.05337398	0.057756	0.017146	0.263444	0.871225	0.060721	0.508028	0.029119	0.024388	0.049707	0.532061	-0.91034	0.542693	0.265445851
C/EBPa/g	CCAAT/enhancer binding protein alpha,	0.05805914	0.386919	0.032044	0.657465	0.734871	0.153808	0.89966	0.024302	0.00559	0.077375	0.620927	-0.68751	0.543441	0.264847805
ATF-a		0.07297201	0.061772	0.02018	0.040375	0.527353	0.030555	0.248677	0.041149	0.026792	0.044116	0.541462	-0.88507	0.546286	0.262580231
L-III BP	pyruvate kinase L gene binding element III (hepatocyte specific)	0.97056135	0.998821	0.943048	0.969784	0.581223	0.924307	1.026348	0.9697	1.014761	0.803886	1.061739	0.086429	0.548996	0.26043087
CEA	carcinoembryonic antigen gene	0.7411114	0.907843	0.933188	0.927229	0.301282	0.895889	1.022996	0.653477	0.793304	0.864196	1.11001	0.150572	0.551881	0.258154423
MBP-1(1)	HIV-EP1; MHC-binding protein 1; PRDII-BF1	0.69658715	0.976902	0.385968	0.89639	0.047701	0.54325	1.000501	0.729108	0.742499	0.575356	1.195491	0.257603	0.553576	0.256823081
COUP-TF	nuclear receptor subfamily 2	0.16327601	0.19662	0.50025	0.94472	0.967892	0.853909	0.952695	0.112175	0.722015	0.833152	1.252885	0.325254	0.55903	0.252565186
LyF-1	LyF-1	0.95341309	0.985931	0.805335	0.953628	0.589882	0.772635	1.026583	0.95458	0.97857	0.824111	1.062565	0.087551	0.560074	0.251754753
oct-1	POU2F1: POU domain, class 2, transcription factor 1	0.02327265	0.017249	0.0013	0	0.608319	0.038841	0.135012	0.029483	0.039127	0.039759	0.434093	-1.20392	0.56083	0.251168548
NRF-1	nuclear respiratory factor 1;	0.02801905	0.171587	0.02232	0.459978	0.630393	0.005276	0.720036	0.007151	0.010149	0.007952	0.571946	-0.80605	0.565136	0.247847199
T3R	c-ErbA; thyroid hormone receptor	0.74980802	0.816025	0.492666	0.879036	0.933953	0.500978	0.94874	0.624054	0.782594	0.708284	0.920744	-0.11913	0.584004	0.23358407
Smad SBE	MADH: MAD, mothers against decapentaplegic homolog	0.06274429	0.069681	0.017038	0.145017	0.710877	0.108268	0.230212	0.022332	0.071602	0.191568	0.620657	-0.68813	0.586545	0.231698645
NFATc	NFATC: nuclear factor of activated T-cells, cytoplasmic,	0.13654309	0.177253	0.113794	0.57064	0.608652	0.448802	0.717958	0.061027	0.201628	0.652622	1.295699	0.373731	0.586619	0.231644236
LH2/Lim1		0.05361896	0.076998	0.025056	0.365661	0.949636	0.059944	0.720003	0.020332	0.013864	0.026796	0.571691	-0.80669	0.58744	0.231036804
CEF1	cTnC (Slow/Cardiac Troponin C)	0.06577586	0.129959	0.156646	0.524054	0.578237	0.206146	0.513694	0.07242	0.073351	0.210957	0.740075	-0.43426	0.589068	0.229834303
AML1	RUNX1: runt-related transcription factor 1 (acute myeloid leukemia 1; aml1 oncogene)	0.35270313	0.098948	0.582623	0.777254	0.021494	0.640933	0.940928	0.155597	0.137083	0.543912	1.319381	0.399861	0.590209	0.228994013
NF-Y	NF-Y, also known as CBF (CCAAT-binding factor), was first identified as a protein that binds to the Y box of MHC (major histocompatibility complex) class II E	0.07095096	0.933063	0.019395	0.794418	0.072157	0.018255	1.024974	0.016454	0.028666	0.010856	0.581595	-0.78191	0.592304	0.227455362
NF-4FA		0.16153056	0.369794	0.576393	0.772133	0.034537	0.784773	0.96634	0.062814	0.158911	0.554587	1.320227	0.400786	0.592629	0.227217182
C/EBPalpha(2)	CCAAT/enhancer binding protein alpha	0.03879796	0.608259	0.026546	0.839587	0.34794	0.146235	0.986052	0.016544	0.001249	0.031988	0.635135	-0.65486	0.597545	0.22362936
LCR-F1	nuclear factor (erythroid-derived 2)-like 1	0.03741997	0.092378	0.027277	0.525198	0.814577	0.104157	0.704816	0.048573	0.010336	0.072727	0.628392	-0.67026	0.60211	0.220324468
AREB6	Atp1a1 Regulatory Element Binding protein 6; Nil-2-a	0.02002673	0.060776	0.017228	0.266413	0.454426	0.020974	0.384558	0.008242	0.025543	0.07723	0.630805	-0.66473	0.602942	0.219724187
GATA-6	GATA binding protein 6 (globin transcription factor 6)	0.08032126	0.154244	0.128963	0.666156	0.108077	0.564903	0.90432	0.055875	0.077753	0.083729	1.482368	0.567904	0.604168	0.218842266
p53		0.15062916	0.631268	0.070454	0.579331	0.426004	0.149277	0.839081	0.087298	0.1067	0.188227	0.73779	-0.43872	0.605847	0.217636778
GAG	amyloid precursor protin (APP) regulatory element	0.41002734	0.249332	0.884918	0.941015	0.32926	0.943307	1.019141	0.142264	0.415216	0.890448	1.211694	0.277026	0.610463	0.214340471
COUP-TF(2)	COUP-TF; EAR3; NR2F1; COUP-alpha	0.03245922	0.052992	0.035295	0.342967	0.185175	0.236312	0.683025	0.029423	0.012053	0.058676	1.57113	0.651803	0.610774	0.214119666
MZF1(2)	myeloid zinc finger 1	0.94226671	0.970955	0.85263	0.89748	0.785943	0.88893	1.011095	0.922642	0.886796	0.852105	1.025239	0.03596	0.612152	0.213140876
IRF-1	IRF1: interferon regulatory factor 1	0.07934136	0.169563	0.299207	0.778317	0.421689	0.763152	0.853128	0.113266	0.09371	0.44998	1.300391	0.378945	0.613256	0.212358202
AFP1	alpha-fetoprotein	0.07664663	0.121241	0.110977	0.741402	0.338419	0.468999	0.981091	0.070056	0.212339	0.192366	1.386096	0.471027	0.614962	0.211151951
Afxh (Foxo4)	myeloid/lymphoid or mixed-lineage leukemia(Member of the forkhead family)	0.92747633	0.980513	0.712724	0.791884	0.907793	0.712788	0.870024	0.967458	0.999866	0.49878	0.937165	-0.09363	0.617396	0.209435877
BZP	a novel serum-responsive zinc finger protein	0.03203052	0.055981	0.015331	0.029423	0.006102	0.033435	0.048443	0.015514	0.002529	0.007879	0.776282	-0.36535	0.61925	0.208134034
HFH-11B/11a	Hepatocyte Nuclear Factor 3/ fork head homolog 11	0.08917099	0.1341	0.501984	0.576743	0.186218	0.844458	0.979247	0.025786	0.017924	0.209177	1.395358	0.480635	0.622501	0.205859607
hTERT-MT-Box	tentative new binding domain	0.92465912	0.944365	0.935355	0.971418	0.912444	0.896342	1.048977	0.922158	1.001865	0.900542	1.017414	0.024907	0.623833	0.204931537
Oct-4/Oct3	Oct-3A; Oct-5; Oct-4A; NF-A3; Oct-3	0.05928401	0.0916	0.166127	0.726146	0.648469	0.759462	0.923295	0.047331	0.036878	0.514211	1.348511	0.431367	0.624214	0.204666664
HFH	HNF-3/Fkh Homolog 1/2; HFH	0.09048774	0.360267	0.308661	0.769735	0.007168	0.658768	0.959937	0.037998	0.027261	0.421695	1.370587	0.454794	0.624752	0.204292601
HFH-1	winged helix/forkhead transcription factor	0.07545238	0.113021	0.14676	0.589575	0.593104	0.734604	0.867309	0.038483	0.168653	0.223592	1.339103	0.421267	0.625717	0.203621744
CdxA/NKX2	Caudal-type homeodomain protein/ cardiac-specific homeo box	0	0.019553	0.040387	0.053206	0.324518	0.050816	0.145764	0.021181	0.008556	0.047783	0.626277	-0.67513	0.626221	0.203272593
MyoG	myogenic factor G	0.01436167	0.048166	0.027521	0.044652	0.060599	0.039294	0.071709	0.020696	0.008775	0.020333	0.823379	-0.28037	0.628168	0.201924
CACC	CACC binding protein	0.30208512	0.368798	0.439087	0.846534	0.087935	0.667507	0.995238	0.112599	0.296712	0.462325	1.239647	0.309929	0.631895	0.199354827
E4F, ATF	E4F transcription factor 1	0.45216308	0.789654	0.330927	0.702035	0.947466	0.634687	0.912466	0.099146	0.554517	0.5946	0.867537	-0.205	0.633932	0.197957608
SAA	amyloid precursor protein (APP) regulatory element containing potential binding site for SP1, AP4, USF, AP1	0.75366638	0.893303	0.685772	0.943684	0.98948	0.788204	1.019543	0.657476	0.836147	0.760134	0.952085	-0.07084	0.634746	0.197399733
C/EBP(a, b & other)	CEBPA: CCAAT/enhancer binding protein (C/EBP), alpha	0.93014044	0.977836	0.887112	0.910202	0.562825	0.19909	0.989069	0.847192	0.965705	0.866157	0.90607	-0.1423	0.637914	0.195237643
Snail	zinc-finger transcription factor Snail	0.27767946	0.087739	0.201747	0.784964	0.68783	0.59727	0.951757	0.207685	0.22511	0.534109	1.233324	0.302552	0.638016	0.195168676
AP4	activator protein 4 binding element	0.89829365	0.970363	0.639913	0.863916	0.801938	0.71049	0.951757	0.879009	0.955868	0.851669	1.041771	0.059038	0.638798	0.194636678
FKHR	forkhead box O1A (rhabdomyosarcoma)	0.05793665	0.072203	0.014465	0.040048	0.175405	0.050913	0.111938	0.047876	0.05118	0.021749	0.78781	-0.34408	0.638877	0.194582721
C/EBPgamma	CCAAT/enhancer binding protein gamma	0.08307723	0.499348	0.312534	0.855416	0.566422	0.66605	1.007944	0.015696	0.011241	0.026941	0.745802	-0.42313	0.643024	0.191772987

Transcription Factor/Cis-element	Description	Normal 1	Normal 2	Normal 3	Normal 4	Normal 5	GBM 1	GBM 2	GBM 3	GBM 4	GBM 5	FC	Log2(FC)	p	-Log10(p value)
NF-Atx	NFATx, a novel member of the nuclear factor of activated T cells family that is expressed predominantly in the Thymus	0.05089361	0.037425	0.033372	0.409087	0.90023	0.100014	0.760567	0.021423	0.009899	0.02233	0.638874	-0.646396	0.655548	0.183395647
NFAT-1	nuclear factor of activated T-cells	0.057844783	0.072888	0.033913	0.482045	0.144213	0.251072	0.900464	0.027332	0.017612	0.02941	1.549985	0.632254	0.65726	0.182262895
ATF-adelta		0.186181197	0.194066	0.164366	0.804607	0.580972	0.407308	0.891648	0.315557	0.065357	0.694741	1.230245	0.298945	0.660297	0.180260728
HFH-8, HNF3.LUN	a new mouse forkhead gene named LUN	0.034847731	0.69083	0.014221	0.639811	0.093381	0.026638	0.876595	0.019241	0.011991	0.016557	0.645597	-0.631295	0.660986	0.179808029
ABF-1	MLC-2 (myosin light chain-2);	0.03227549	0.142787	0.129722	0.70318	0.076599	0.517971	0.965904	0.002091	0.008431	0.082422	1.453876	0.539905	0.673531	0.171642643
Stat5	STAT5: signal transducer and activator of transcription 5	0.283895703	0.36419	0.056342	0.343567	0.122796	0.382579	0.078179	0.2032	0.318977	0.370971	1.156404	0.209646	0.675199	0.170568289
MTF	MRE-binding transcription factor-1	0.519868122	0.476868	0.428793	0.727671	0.549284	0.637082	0.948605	0.469184	0.338681	0.548088	1.088494	0.122334	0.687223	0.162902194
Skn	octamer-binding site in epidermis (POU domain factor)	0.253947478	0.790027	0.38307	0.907369	0.277065	0.712076	1.018805	0.027877	0.727323	0.567622	1.169339	0.225693	0.688251	0.162253315
MEF-3	myelin gene expression factor 3	0.098970005	0.113613	0.073705	0.508689	0.452135	0.473337	0.813234	0.121478	0.153696	0.038996	1.283558	0.360149	0.692068	0.159851147
DE I	Rat DE1 element from albumin gene	0.061182571	0.254998	0.091826	0.694925	0.734129	0.310045	0.92249	0.022968	0.007963	0.114919	0.75032	-0.414421	0.693112	0.159196751
Tax/CREB	TAX &CREB complex-responsive element	0.100194881	0.205742	0.268382	0.570668	0.038413	0.356329	0.950483	0.03221	0.095084	0.138011	1.328476	0.409772	0.696029	0.157372704
NFkB	NFKB: nuclear factor of kappa light polypeptide gene enhancer in B-cells 1	0.223386814	0.326454	0.151879	0.433606	0.230122	0.447281	0.045861	0.194988	0.288968	0.609523	1.161979	0.216584	0.696548	0.157048663
E2	BPV-1, bovine papilloma virus type 1	0.482999345	0.900745	0.210469	0.929872	0.610226	0.348917	0.980923	0.672263	0.154414	0.588101	0.875669	-0.191543	0.699791	0.155031676
SIF1	Sucrase-isomaltase (SI) is an enterocyte-specific gene which exhibits a complex pattern of expression during intestinal development and in the adult intestinal mucosa SIF1SI	0.022078395	0.079613	0.017173	0.23146	0.790502	0.118367	0.606422	0.021847	0.007369	0.026796	0.684417	-0.547052	0.707648	0.15018262
C/EBPalpha(1)	CCAAT/enhancer binding protein alpha	0.514784885	0.787505	0.346827	0.858167	0.22458	0.639801	0.969994	0.540907	0.191605	0.729816	1.124552	0.16935	0.710147	0.148651644
Smad 3/4	MADH3/4: MAD, mothers against decapentaplegic homolog3/4	0.640916522	0.83726	0.671903	0.875985	0.98824	0.829569	0.983638	0.232017	0.865999	0.825091	0.93075	-0.103534	0.715225	0.145557302
Freac-7	forkhead box L1	0.024987476	0.055296	0.036243	0.366233	0.560063	0.131961	0.552482	0.015878	0.028135	0.033513	0.730679	-0.452691	0.71598	0.14509916
ACF	albumin ccaat-binding factor	0.040390296	0.113768	0.019449	0.679805	0.477223	0.030846	0.862615	0.015363	0.009149	0.020406	0.705212	-0.503872	0.724068	0.140220587
MZF1(1)	myeloid zinc finger 1	0.771182116	0.834022	0.865822	0.954146	0.392122	0.947871	1.026114	0.420278	0.416715	0.713839	0.923381	-0.115002	0.725195	0.139545016
Pit 1	POU1F1: POU domain, class 1, transcription factor 1 (Pit1, growth hormone factor 1)	0.069756705	0.238372	0.275343	0.673403	0.309323	0.492498	0.925909	0.049088	0.152291	0.275878	1.210361	0.275437	0.729865	0.136757473
URE		0.374781524	0.461425	0.081939	0.124557	0.456096	0.106455	0.635287	0.263166	0.205813	0.054827	0.844375	-0.244045	0.731098	0.13602459
Freac-2 (2)	forkhead box F2 (Fork head REIated	0.573823922	0.840965	0.862219	0.954636	0.741533	0.884496	1.032517	0.134204	0.862533	0.758355	0.924224	-0.113686	0.731123	0.13600962
RSRFC4	Ras-responsive transcription element	0.058457221	0.147613	0.615426	0.945782	0.48298	0.860803	1.020649	0.016029	0.130745	0.678039	1.202647	0.266213	0.731184	0.135973394
Pax-6	Pax-6 plays an essential role in the development and function of glucagon-producing cells in both pancreatic and	0.111371877	0.073479	0.613259	0.869419	0.454146	0.876339	0.998087	0.026908	0.087434	0.573504	1.207665	0.27222	0.732984	0.134905626
CD28RC	T-cell accessory molecule CD28	0.057385454	0.186812	0.073407	0.468941	0.620148	0.128465	0.851787	0.015817	0.024856	0.054464	0.764481	-0.387448	0.743788	0.128550885
AF1, ARP1., NF-BA	NR2F2: nuclear receptor subfamily 2, group F, member 2	0.168267381	0.262595	0.190424	0.742791	0.880536	0.48596	0.941197	0.148294	0.084655	0.218836	0.837088	-0.256548	0.745305	0.127665824
Beta-response element	Beta-response element	0.796996383	0.936208	0.607435	0.807495	0.752213	0.811767	0.945219	0.447065	0.988063	0.89259	1.047267	0.066629	0.747245	0.12653717
HNF1a/b/c	hepatocyte nuclear factor-1	0.04173766	0.030264	0.042202	0.543588	0.773556	0.209545	0.81377	0.017726	0.00534	0.019208	0.744466	-0.425722	0.74809	0.126045936
Nkx-2.5	cardiogenic homeodomain	0.022752077	0.025282	0.023078	0.350568	0.4203	0.241005	0.380937	0	0.016425	0.010203	0.770292	-0.376523	0.751645	0.123987016
X2BP	X2 binding protein, X2BP	0.031999893	0.127188	0.01758	0.337737	0.042437	0.104416	0.62	0.020665	0.004309	0.01899	1.379644	0.464296	0.757198	0.120790302
CTCF	CCCTC binding factor	0.932008373	0.97055	0.860946	0.921045	0.971582	0.862616	1.01837	0.852283	1.002302	0.852105	0.985298	-0.021368	0.758795	0.119875353
PU.1	Spi-1, NF-JB, B1: acts as an activator or as a repressor; after phosphorylation at Ser-148 by CK II, PU.1 recruits NF-EM5 to bind to DNA resulting in pronounced transcriptional activation; PU.1 interferes with the commitment of erythroblast to differentiate; activated by proviral integration of SFFV (anemia- or polycythemia-inducing strains) in 95% of leukemic cell clones	0.140983262	0.177316	0.134949	0.846071	0.677366	0.714374	0.975157	0.103418	0.117005	0.427214	1.182367	0.241678	0.759794	0.119304331
GKLF	gut-enriched Krueppel-like factor; EZF; epithelial zinc-finger	0.115168994	0.216422	0.126011	0.340543	0.846055	0.236021	0.773005	0.088419	0.039564	0.213389	0.821311	-0.284	0.7632	0.117361846
LR1	LR1 is a 106-kDa sequence-specific DNA-binding protein first identified as a potential regulator of immunoglobulin class switch recombination in B lymphocytes	0.033377879	0.62473	0.018067	0.629404	0.018841	0.023596	0.890005	0.015363	0.014114	0.026578	0.732136	-0.449816	0.763988	0.116913526
transferrinBP	transferrin binding protein	0.344925165	0.439724	0.216266	0.657928	0.194831	0.619895	0.948941	0.296467	0.138364	0.126356	1.149082	0.200481	0.765781	0.11589534
FAST-1	FOXH1: forkhead box H1	0.388163298	0.5208	0.611037	0.762597	0.271809	0.908027	0.002213	0.302709	0.73413	0.914557	1.120274	0.163852	0.766434	0.115525409
TxREF, NF III	Tax-responsive element (TxRE) -binding factor	0.219957161	0.471638	0.363567	0.83926	0.886528	0.629573	0.966574	0.313678	0.217803	0.365198	0.896394	-0.157795	0.768093	0.114586025
c-Ets-1	c-Ets-1A; p54c-Ets-1; p54; Ets1; c-Ets-1 54	0.064122274	0.053615	0.035295	0.309104	0.398282	0.165137	0.449294	0.029998	0.029509	0.017537	0.80365	-0.31536	0.770165	0.11341647
ISRE (1)	interferon-a stimulated response element	0.069572974	0.507007	0.091989	0.779434	0.27701	0.205984	0.974587	0.042725	0.080595	0.091136	0.808705	-0.306315	0.773092	0.111769016
MAZ	MYC-associated zinc finger protein (purine-binding transcription factor)	0.672303977	0.811822	0.807286	0.865768	0.808923	0.829472	1.00972	0.521727	0.738908	0.739946	0.968148	-0.046701	0.774352	0.111061578
Brn-3	POU4F1: POU domain, class 4, transcription factor 1	0.783063416	0.928984	0.586496	0.8361	0.728396	0.707869	0.877299	0.804498	0.827372	0.741652	1.02476	0.035286	0.774698	0.11086754
PPARa	peroxisome proliferator activated receptor	0.134583283	0.327045	0.134462	0.703752	0.126436	0.253305	0.7195	0.093934	0.075068	0.528154	1.170852	0.227559	0.779225	0.108337009
PEBP	polyoma enhancer binding protein	0.11124939	0.100349	0.064928	0.594533	0.27757	0.232396	0.800629	0.127114	0.050274	0.177915	1.208682	0.273435	0.780477	0.10764009
HNF-4 & COUP-TF	HNF-4 and COUP-TF	0.109381453	0.054207	0.854553	0.950359	0.345551	0.889934	1.011296	0.081662	0.058768	0.660247	1.167609	0.223557	0.784001	0.105683644
TCF/LEF	T cell factor/lymphocyte enhancer binding	0.029121434	0.187123	0.018961	0.233285	0.454241	0.057775	0.599784	0.009909	0.021983	0.036418	0.786652	-0.346202	0.784141	0.105606026
ARP	COUP-beta; apolipoprotein AI regulatory protein; NR2F2	0.388500139	0.246436	0.447375	0.837381	0.654716	0.480652	0.917193	0.225835	0.345269	0.382155	0.91326	-0.130902	0.784581	0.105362342
RFX1/2/3	a transactivator of hepatitis B virus enhancer I, belongs to a novel family of homodimeric and heterodimeric DNA-binding proteins	0.172891289	0.614144	0.039358	0.792402	0.625753	0.163292	0.893793	0.364918	0.316697	0.233904	0.878842	-0.186324	0.786763	0.104156243
CREB2	cyclic AMP response element binding protein 2. An ATF/CREB transcription factor that can negatively regulate transcription	0.347129942	0.739277	0.076955	0.468559	0.149181	0.103898	0.623687	0.34701	0.383865	0.115826	0.883883	-0.178072	0.793177	0.100630037
TFE3		0.141289481	0.141635	0.102038	0.685689	0.410643	0.386334	0.859431	0.088964	0.092211	0.297845	1.164377	0.219558	0.793654	0.100368849
ORE	osmotic response element	0.114740287	0.339562	0.188718	0.652752	0.327868	0.526483	0.859565	0.073208	0.055302	0.343739	1.144525	0.194749	0.796935	0.098577133
WT1 (3)	Wilms tumor 1	0.243627895	0.414722	0.188664	0.580857	0.720926	0.427084	0.853698	0.15387	0.079971	0.41171	0.89647	-0.157673	0.798653	0.09764161
AIC, CBF	ApoA-I gene promoter c region, CCAAT-binding factor	0.36106291	0.418116	0.235146	0.617935	0.918311	0.434755	0.854905	0.263257	0.43211	0.359316	0.919144	-0.121637	0.79889	0.097513103
SRF, SAP	Serum Response Factor	0.029243922	0.129305	0.023756	0.475125	0.009462	0.122477	0.727143	0.002697	0.007713	0.020878	1.320917	0.40154	0.801789	0.095939772
CYP1A1	cytochrome P450-c	0.317426692	0.44729	0.75089	0.839288	0.451424	0.912461	0.998423	0.230744	0.106419	0.82992	1.096799	0.133299	0.802798	0.095393643
HOXD9,10	HOXD9,10	0.646214112	0.875214	0.415141	0.850893	0.496598	0.523085	0.986723	0.525939	0.797801	0.61595	1.050375	0.070905	0.803078	0.095242067
MTB-Zf	cis- regulatory element (MTE) binding protein	0.390827404	0.397349	0.115013	0.474308	0.08099	0.160023	0.666364	0.389159	0.305081	0.102065	1.112586	0.153917	0.804052	0.094715722
MDBP(1)	methylated DNA-binding protein	0.122610117	0.135345	0.048974	0.739822	0.035871	0.185075	0.892352	0.097206	0.089619	0.076032	1.237998	0.308009	0.808303	0.092425835
HFH-8	forkhead box F1a (HNF-3/Fkh Homolog-8)	0.034725243	0.106763	0.286097	0.696069	0.884816	0.45861	0.82946	0.016332	0.07054	0.360514	0.864069	-0.210782	0.811961	0.090464858
Stat1 (p84/p91)	STAT1: signal transducer and activator of transcription 1, 84kDa/91kDa	0.210770589	0.257147	0.1515	0.65831	0.523012	0.415205	0.33169	0.082813	0.277414	0.541225	0.915374	-0.127567	0.812065	0.090409076
NF-E2(2)		0.279455527	0.437109	0.455312	0.859802	0.677456	0.697641	0.941565	0.095328	0.25362	0.500777	0.918718	-0.122306	0.815262	0.088702548
NFE-6/CP1	A highly conserved enhancer downstream of the human MLC1/3 locus is a target for multiple myogenic determination factors	0.172891289	0.385175	0.155617	0.785945	0.399231	0.389829	0.896207	0.057875	0.185203	0.144874	0.881576	-0.181843	0.817564	0.087478502
p55	p55kDa was induced by TPA and interaction with the cdc2 promoter in R-box dependent	0.101511623	0.12918	0.343008	0.712224	0.201898	0.468579	0.878137	0.033968	0.031289	0.305905	1.154626	0.207425	0.817702	0.087404927
AAF	an IFN-gamma-regulated DNA-binding factor	0.04173766	0.219473	0.020722	0.639348	0.293567	0.059976	0.890307	0.008363	0.010398	0.001416	0.798833	-0.324034	0.819394	0.086507155
NF-Atp	NFII-a; NF-ATc2; NF-IL2E; NF-AT1;	0.188845303	0.424904	0.508539	0.894919	0.623523	0.73305	0.996478	0.033695	0.11738	0.505606	0.903617	-0.146216	0.819494	0.086454433
Stat5/Stat6	STAT5/6: signal transducer and activator of transcription 5/6	0.037052508	0.117909	0.534678	0.79785	0.462399	0.780856	0.906029	0.027029	0.031757	0.473291	1.137994	0.186493	0.821271	0.08551374
NPAS2	neuronal PAS domain protein 2	0.015341576	0.021826	0.054256	0.523972	0.471608	0.469323	0.654932	0.00803	0.012584	0.14393	1.185643	0.245669	0.821928	0.085166025
TEF1(2)	activator; function depends on a highly limiting cofactor. interference with TEF-1 / TFIID interaction by a B-cell specific factor	0.986852209	1.018841	0.991426	1.041515	0.957302	0.976418	1.046832	0.987457	1.032529	0.976355	1.004735	0.006815	0.825169	0.083456871
PPUR(2)	purine-rich sequences binding sequence	0.080321263	0.141946	0.053579	0.624473	0.425157	0.13086	0.787521	0.042573	0.020516	0.13852	0.844971	-0.243026	0.826264	0.082881415
PTF1-beta	pancreas specific transcription factor	0.110575708	0.153715	0.129559	0.696805	0.248806	0.364551	0.888999	0.066117	0.074725	0.158817	1.159577	0.213599	0.827052	0.082467318
E12/E47	TCF3; E2A.E12; E2A-E12; Pan-2 (rat); E2-	0.460798462	0.310917	0.637312	0.685335	0.795133	0.807041	0.93077	0.309467	0.053428	0.583961	0.929113	-0.106074	0.827628	0.082164687
CP1	CBF; NF-Y	0.077932754	0.050252	0.383097	0.74824	0.954143	0.734571	0.934425	0.050482	0.028197	0.761041	1.133286	0.180512	0.828013	0.081963006
Ikaros	Ikaros protein (zinc-finger protein)	0.10901399	0.066007	0.103989	0.176347	0.474944	0.188668	0.604511	0.143901	0.060173	0.071021	1.148311	0.199513	0.830889	0.080457169
GATA-3	GATA binding protein3 (globin transcription factor 3)	0.060968218	0.041192	0.315649	0.293221	0.657375	0.622355	0.676522	0.031604	0.038596	0.194981	1.142978	0.192797	0.832955	0.079378264
RVF		0.095019779	0.093437	0.069696	0.637387	0.39107	0.461361	0.895335	0.046634	0.028572	0.072945	1.169622	0.226042	0.834787	0.078424356
HiNF-D3	histone H3	0.047249603	0.100038	0.062599	0.739114	0.094224	0.288359	0.928491	0.020544	0.010617	0.029047	1.224145	0.291775	0.837478	0.077026662
GATA-4	GATA binding protein 4 (globin transcription factor 4)	0.120956534	0.577934	0.221981	0.807849	0.498344	0.450421	0.992019	0.048633	0.20116	0.323334	0.905033	-0.143957	0.840582	0.075419736
LF-B2	liver-specific factors	0.043973059	0.055888	0.038925	0.206396	0.654482	0.173552	0.578027	0.02115	0.018798	0.045132	0.836942	-0.2568	0.842528	0.074415563
HNF-4alpha2/1	hepatocyte nuclear factor-4	0.170564024	0.205586	0.15605	0.748649	0.591121	0.404524	0.900029	0.077026	0.07079	0.21909	0.892887	-0.163451	0.844018	0.073648377
MBP-1(2)	myc-promoter binding protein	0.04881132	0.049007	0.359314	0.592735	0.230472	0.536388	0.910187	0.020635	0.011866	0.011292	1.164041	0.219142	0.846544	0.072350323
VDR(DR-3)	VDR: vitamin D (1,25- dihydroxyvitamin D3) receptor	0.382069538	0.47142	0.482427	0.853018	0.865954	0.797558	0.908343	0.255288	0.492657	0.754578	1.050259	0.070745	0.84961	0.070780178
CP1, CTF, CBTF	CCAAT-box-binding transcription factor	0.032887929	0.159537	0.031882	0.438183	0.002255	0.032302	0.714639	0.011787	0.032944	0.027595	1.232453	0.301532	0.851465	0.069833443
MZF1	zinc finger protein 42 (myeloid-specific retinoic acid- responsive)	0.040880246	0.048727	0.118236	0.284857	0.783788	0.324092	0.658217	0.015999	0.025075	0.07556	0.860911	-0.216064	0.853251	0.068923027
NCAM binding protein	neural cell adhesion molecule	0.176320943	0.122237	0.629674	0.794118	0.989868	0.888121	0.973648	0.121417	0.148325	0.817938	1.087468	0.120973	0.856145	0.067452477
KPF1	Keratinocyte-Specific Transcription Factor	0.123498152	0.280218	0.069506	0.739577	0.40573	0.192422	0.792583	0.231229	0.077472	0.165062	0.901293	-0.149932	0.859678	0.065664382
HiNF-A	A nuclear protein with affinity for the 5' flanking region of a cell cycle dependent human H4 histone gene	0.079402606	0.265771	0.243055	0.670351	0.824526	0.740883	0.936604	0.028665	0.009899	0.15003	0.895816	-0.158725	0.860888	0.065053279
PRE	Progesterone receptor	0.188875925	0.18675	0.144484	0.681821	0.684663	0.398698	0.606288	0.103539	0.180832	0.459457	0.926969	-0.109406	0.863612	0.063681328
RREB (1)	Ras-responsive transcription element	0.574528226	0.809954	0.398862	0.93908	0.835462	0.612289	0.959132	0.441641	0.675519	0.754796	0.967815	-0.047197	0.864521	0.063224546
PEPCK promoter GR	PEPCK (phosphoenolpyruvate carboxykinase); glucocorticoid receptor; GR-DBD (GR DNA-binding domain); GCR	0.061703143	0.073137	0.234008	0.805288	0.833016	0.651356	0.986421	0.026332	0.015519	0.098362	0.885827	-0.174903	0.865641	0.062662252
NF-1/L	NF1/L; RPF-A (hamster); NF-1A1.1 (chick); NF-I/L	0.184803211	0.211129	0.543454	0.81703	0.799961	0.861418	0.967647	0.055482	0.052023	0.830937	1.082589	0.114486	0.868352	0.061304164
PUR	Pur factor	0.172952533	0.123825	0.205295	0.6755	0.401803	0.652359	0.819972	0.096206	0.068167	0.101884	1.100807	0.138562	0.871233	0.059865732
Gfi-1	growth factor independent 1	0.802661436	0.907501	0.688562	0.866667	0.302348	0.645562	0.906331	0.851041	0.953121	0.34265	1.036708	0.05201	0.871376	0.059794421
MDBP(2)	methylated DNA-binding protein	0.057416076	0.060496	0.039439	0.46025	0.159323	0.142545	0.717656	0.020787	0.012366	0.011728	1.164954	0.220272	0.874899	0.058041917
IRF-1, IRF-2	interferon regulatory factor 1/2 binding element	0.099674309	0.051747	0.124358	0.443386	0.706093	0.270945	0.841428	0.059178	0.03463	0.058894	0.887611	-0.172001	0.875661	0.057664129
HOX4C	homeo box4C	0.075850465	0.08721	0.139473	0.626026	0.930965	0.459839	0.906868	0.033119	0.061859	0.207072	0.897411	-0.15616	0.876161	0.05741617
CETP-CRE	CETP (Cholesteryl ester transfer protein)	0.056528041	0.139766	0.36698	0.748622	0.371636	0.648669	0.898688	0.045967	0.090119	0.166042	1.098575	0.135633	0.877588	0.056709272
PRDI-BFc	an inducible factor that binds to a positive regulatory element of the human beta-interferon gene	0.091008309	0.07762	0.03218	0.173731	0.36661	0.09189	0.467765	0.042573	0.029603	0.028249	0.890616	-0.167124	0.879234	0.055895679
kBF-A	kappa immunoglobulin enhancer binding	0.716613877	0.421136	0.880909	0.929736	0.647965	0.911976	1.028226	0.427399	0.454874	0.89426	1.033471	0.047498	0.880329	0.055354853
HSE	heat shock transcription factor	0.106196775	0.154992	0.323938	0.821144	0.812443	0.766615	0.930838	0.024817	0.125499	0.549758	1.080593	0.111823	0.883265	0.053908881
KLF	Kruppel-like factor 15	0.505659557	0.727072	0.797615	0.816648	0.060443	0.889772	1.001641	0.296255	0.551644	0.319848	1.052184	0.073387	0.884452	0.053325672
ETF	DNA Binding Factor That Represses Transcription	0.787932299	0.886204	0.783936	0.838933	0.731835	0.71172	1.004591	0.773621	0.687791	0.804903	0.988529	-0.016645	0.885069	0.05302268

Transcription Factor/Cis-element	Description	Normal 1	Normal 2	Normal 3	Normal 4	Normal 5	GBM 1	GBM 2	GBM 3	GBM 4	GBM 5	FC	Log2(FC)	p	-Log10(p value)
LF-A1(2)	Liver-specific transcription factor	0.05720172	0.057818	0.02018	0.312754	0.485002	0.081695	0.617318	0.029816	0.0242	0.073127	0.885524	-0.1754	0.887004	0.052074519
Pax2	paired box gene 2(gene5/gene8)	0.66434228	0.975345	0.491284	0.924886	0.002866	0.34089	1.008916	0.644538	0.529817	0.379867	0.949425	-0.07487	0.888028	0.051573406
Freac-4	forkhead box D1	0.0331329	0.064481	0.163472	0.752327	0.44829	0.438283	0.91726	0.031119	0.054802	0.174538	1.105562	0.14478	0.888983	0.051106575
Isl-1	Islet-1	0.03359223	0.064793	0.098869	0.759628	0.872432	0.54367	0.921317	0.016787	0.005933	0.162375	0.902022	-0.14877	0.892093	0.049589678
HEN1	HEN1 and JEN2 encode a distinct subgroup of bHLH proteins. bHLH pretein by HEN1 and HEN2 may severs important regultory functions in the developing nervious system	0.04516731	0.060216	0.066581	0.720997	0.872986	0.537391	0.874215	0.035392	0.022795	0.458803	1.092103	0.127109	0.897192	0.047114818
PCF	Promoter-linked coupling element	0.14689329	0.249954	0.059728	0.747287	0.37884	0.264731	0.862615	0.069875	0.075505	0.186375	0.921905	-0.11731	0.899715	0.045894881
Stat3	STAT3: signal transducer and activator of transcription 3	0.42230672	0.815714	0.302566	0.709936	0.757919	0.599827	0.490998	0.434853	0.809074	0.752	1.02603	0.037073	0.902689	0.044461898
LyF-1	LyF-1	0.15926454	0.489447	0.16889	0.667028	0.547729	0.330759	0.842501	0.16702	0.517576	0.266619	1.045325	0.063952	0.909723	0.041090669
Freac-2 (1)	forkhead box F2 (mouse)	0.01757697	0.077309	0.106833	0.394457	0.207887	0.101018	0.64293	0.015666	0.038408	0.084564	1.097658	0.134429	0.910178	0.040873527
MHC gene promoter W	MHC class II Ebeta	0.05827349	0.109036	0.820531	0.914479	0.84626	0.827853	1.016124	0.040301	0.017612	0.683594	0.940661	-0.08825	0.910475	0.040731978
LXRE1	nuclear receptor subfamily 1, group H,	0.15016983	0.5312	0.05813	0.746251	0.126534	0.244987	0.92896	0.122962	0.072164	0.123851	0.925968	-0.11097	0.911897	0.040054404
GBF1/2/3/HY5	G-box binding factor 1	0.3940427	0.490848	0.436378	0.688005	0.58478	0.617144	0.976129	0.177686	0.273948	0.635157	1.033157	0.04706	0.912798	0.039625452
MyTI	myelin transcription factor I	0.03199989	0.053708	0.116503	0.784501	0.568193	0.55215	0.864727	0.025786	0.026948	0.206999	1.078272	0.108721	0.916283	0.037970385
Pax3	paired box gene 3	0.19622518	0.503738	0.120485	0.830733	0.379976	0.297292	0.945689	0.135628	0.316572	0.232851	0.949228	-0.07517	0.916588	0.037825636
Pax5(2)	Pax5 is required for the early stages of B-cell differentiation, Pax-5DNA-binding transcription factor	0.2212739	0.242855	0.078418	0.555983	0.57432	0.428217	0.826811	0.103994	0.066856	0.33557	1.052962	0.074453	0.918828	0.036765839
LF-A2	liver-specific factors stimulate in vitro transcription from the human alpha1-antitrypsin promoter	0.09097769	0.078866	0.091745	0.645342	0.436821	0.209868	0.917495	0.067481	0.056176	0.191495	1.073498	0.10232	0.922941	0.034826015
p53(2)	Tumor protein p53	0.25578479	0.341897	0.23501	0.743854	0.810468	0.549561	0.862079	0.350888	0.151854	0.542859	1.029421	0.041833	0.936653	0.028421258
GATA	GATA: GATA binding protein (globin transcription factor)	0.07532989	0.104864	0.140719	0.547293	0.668129	0.508681	0.759696	0.102661	0.102485	0.130895	1.044315	0.062557	0.942504	0.025716788
Fra-1/JUN	Fos-related antigen	0.4010245	0.464944	0.787566	0.886283	0.498907	0.844134	1.026147	0.178717	0.218522	0.845497	1.024449	0.034848	0.942844	0.025560293
beta M-globin factor B1	beta M-globin factor B1	0.05995769	0.057756	0.088251	0.35381	0.642122	0.359599	0.724998	0.021968	0.012022	0.026869	0.95304	-0.06939	0.951873	0.021420825
GATA1(2)		0.04378933	0.056697	0.050003	0.509288	0.438097	0.174977	0.614032	0.030301	0.019954	0.215786	0.960992	-0.0574	0.955903	0.019586377
EVI-1	ecotropic viral integration site 1(zinc finger oncogene)	0.25486614	0.720596	0.341599	0.752763	0.276475	0.659447	0.94656	0.057421	0.306018	0.428703	1.022099	0.031535	0.957286	0.01895843
Elf-1	E74-like factor 1,a novel Ets family member	0.01846501	0.022199	0.017228	0.383669	0.275442	0.051108	0.679639	0.011242	0.000906	0.015649	1.057936	0.081252	0.958161	0.01856137
Elk1	member of ETS oncogene family	0.03738935	0.054362	0.202235	0.73549	0.810804	0.603549	0.937341	0.015635	0.032226	0.188046	0.965503	-0.05065	0.96017	0.017651904
AP2	activator protein 2	0.14827128	0.155708	0.194596	0.443169	0.220794	0.188668	0.664654	0.054512	0.049306	0.173594	0.972644	-0.04002	0.960891	0.017325654
v-rel 50-55K	NF-kappaB 50-55K protein	0.50351602	0.497791	0.860539	0.941696	0.577433	0.967582	0.98575	0.265318	0.237445	0.876469	0.985681	-0.02081	0.961542	0.017031929
SRY	testis determining factor binding domain	0.04639219	0.208358	0.028333	0.626735	0.259935	0.150863	0.913505	0.010363	0.04175	0.101847	1.041527	0.0587	0.96258	0.016563024
Cdx2	caudal type homeo box transcription factor 2	0.03570514	0.357309	0.479339	0.826565	0.521913	0.727742	1.003518	0.009484	0.026105	0.510435	1.02542	0.036215	0.962588	0.016559418
TR	thyroid hormone receptor	0.72194209	0.879417	0.497921	0.875603	0.779848	0.634848	0.931977	0.492698	0.968109	0.75407	1.007184	0.010327	0.963262	0.01625561
CDP	CCAAT displacement protein	0.03916542	0.022324	0.160953	0.519232	0.777838	0.116942	0.726004	0.018908	0.119441	0.584433	1.030415	0.043225	0.965285	0.015344465
HNF-1A	hepatocyte nuclear factor 1	0.23238966	0.77667	0.641294	0.884294	0.555307	0.813612	1.003451	0.151658	0.321381	0.757047	0.986147	-0.02013	0.966058	0.014996723
LSF		0.86182296	0.642384	0.820315	0.77908	0.835687	0.944148	0.955176	0.675869	0.455623	0.886345	0.994383	-0.00813	0.967001	0.014572883
TCE	Transforming Growth Factor ß1control	0.05147543	0.213713	0.043421	0.602515	0.212266	0.233076	0.801098	0.014332	0.022514	0.088413	1.032084	0.04556	0.968884	0.01372827
Thy-1binding protein	Thy-1binding protein	0.81438963	0.9009	0.821723	0.943603	0.335148	0.946091	0.990813	0.854859	0.103265	0.884275	0.990445	-0.01385	0.971528	0.012544666
Pax-5	PAX5: paired box gene 5 (B-cell lineage specific activator protein)	0.8560048	0.946109	0.490851	0.731949	0.568793	0.722499	0.183244	0.794408	0.934572	0.930351	0.992033	-0.01154	0.972771	0.011989512
HiNF	histone gene transcription factors	0.43832198	0.984561	0.278648	0.902356	0.477682	0.454887	1.038015	0.149325	0.87646	0.528227	0.988754	-0.01632	0.974443	0.011243614
c-Myc-responsive region	c-myc responsive region	0.03800179	0.06856	0.274504	0.639484	0.60455	0.595263	0.785007	0.04227	0.015582	0.218436	1.019358	0.02766	0.975663	0.010700267
XBP-1	X-box binding protein 1	0.48897062	0.981883	0.187905	0.910121	0.180025	0.326843	1.014313	0.171081	0.782375	0.488105	1.0123	0.017638	0.977226	0.01000482
c-myb binding protein	c-myb binding protein	0.82421926	0.96012	0.757553	0.90459	0.619646	0.694048	0.948002	0.86304	0.763514	0.806864	1.002297	0.00331	0.980339	0.008623682
LF-A1	liver-specific TF	0.66112698	0.945393	0.447619	0.845881	0.429107	0.470747	1.027421	0.406158	0.871995	0.570563	1.005334	0.007675	0.982665	0.007594626
Ets-1/PEA3	ETS-domain transcription factor pea3	0.17779079	0.474471	0.040062	0.206451	0.462753	0.126167	0.185122	0.092449	0.356823	0.615296	1.010524	0.015104	0.982757	0.007553817
RIPE3a1	rat insulin promoter element 3(RIPE3)can confer either positive regulation in insulin-	0.1376761	0.426803	0.128476	0.521521	0.23398	0.259746	0.764758	0.106933	0.034599	0.29799	1.01075	0.015426	0.983942	0.007030395
TTF-1(1)	Thyroid-specific enhancer. Homog of Drosophila NK2 factor, and mammalian Nkx family; site in promoters of eight thyroid-specific genes; also regulates pulmonary-	0.23144038	0.36995	0.080639	0.402848	0.336087	0.294703	0.629286	0.233259	0.198474	0.074216	1.006315	0.009082	0.987372	0.005519378
EGF binding protein	epidermal growth factor	0.04280943	0.045644	0.05989	0.624991	0.517354	0.342897	0.883803	0.013999	0.008556	0.029265	0.990572	-0.01367	0.991154	0.003858908
XBP1, X2BP	X-box-binding protein 1	0.02973387	0.154525	0.015142	0.526152	0.295956	0.064475	0.871633	0.018544	0.005059	0.053011	0.9914	-0.01246	0.992949	0.003073232
CD28RC, NF-IL2B	T-cell accessory molecule CD28	0.02161907	0.254811	0.021209	0.396991	0.162109	0.020391	0.805389	0.000182	0.008993	0.017283	0.994746	-0.0076	0.996004	0.001738879
ACPBP(CRY-alphaA)	alphaA-crystallin	0.32030515	0.302915	0.328381	0.644715	0.557216	0.604197	0.844479	0.20926	0.131525	0.365489	1.000658	0.000949	0.99853	0.000638968
